# Research on Cancer Prediction Based on Feature Optimization and Multimodal Fusion

**DOI:** 10.1002/hcs2.70040

**Published:** 2025-12-01

**Authors:** Jiawei Xu, Guodong Bao, Hansen Chen, Yifan Zhao, Mengqiang Yu, Jiqiang Shang, Yanxuan Luo, Hongbo Ge, Weiqi Hu, Wenhua Zhang, Xiangyi Zan, Zhixuan Yu, Minjie Ma, Xiong Cao, Menghao Guo, Chenxi Shi, Pengfei Cao, Lin Cheng

**Affiliations:** ^1^ School of Information Science and Engineering, Lanzhou University Lanzhou China; ^2^ The People's Government of Jiayuguan Municipality Jiayuguan China; ^3^ Geriatric Department The Second Hospital of Lanzhou University Lanzhou China; ^4^ Information Center The First Hospital of Lanzhou University Lanzhou China; ^5^ Department of Thoracic Surgery The First Hospital of Lanzhou University Lanzhou China; ^6^ Electronic Information Science and Technology Lanzhou University Lanzhou China

**Keywords:** fiber optic signal, low‐rank multimodal fusion, lung cancer, prediction, sound signal

## Abstract

**Background:**

Current lung cancer initial diagnosis relies on experienced doctors combining imaging and biological indicators, but uneven medical resource distribution in China leads to delayed early diagnosis, affecting prognosis. Existing methods struggle with large‐scale screening, multitracking, and over‐reliance on single‐modality data, ignoring the potential of multisource complementary information. Key technical challenges—effective data collection, multimodal feature extraction/fusion, and AI model construction—limit clinical application. Thus, exploring AI, new sensors, and existing data for efficient, fast, accurate, and radiation‐free preliminary diagnosis is crucial for timely treatment and improved outcomes.

**Methods:**

This study collected hematological data, and used fiber‐optic vibration sensors and audio sensors to capture heterogeneous signals of patients' lung respiration. Fiber‐optic respiratory frequency, audio‐respiratory rhythm, and hematological leukocyte‐related features were extracted, optimized as multimodal inputs. The SCCA‐LMF fusion method generated fusion samples, which were input into an improved stacking ensemble learning model (including SVM, XGBoost, etc.) for binary classification.

**Results:**

The experiment included 360 actual samples (lung cancer: nonlung cancer = 3.6:1) with complete data of 55–65‐year‐old males and females. Predictive accuracy, sensitivity, specificity, and F1 score reached 97.70%, 95.75%, 99.64%, and 99.64%, respectively, outperforming existing independent LMF and TFN methods. This model effectively integrates respiratory vibration, audio signals, and routine blood tests. A multimodal feature grading fusion strategy was designed for 3D data analysis to comprehensively understand patient health and enhance prediction capabilities. All data and results are reproducible.

**Conclusion:**

This study demonstrates the method's potential for lung cancer preliminary identification, bridging medicine and engineering to improve healthcare outcomes.

## Graphical abstract

This study collected patient respiratory signals through fiber optic vibration sensors and audio sensors, combined with hematological indicators, and achieved lung cancer prediction through SCCA‐LMF multimodal fusion method and improved Stacking ensemble learning model. The accuracy was 97.70%, sensitivity was 95.75%, and specificity was 99.64% in 360 samples, significantly better than traditional single modal or single fusion methods, providing a noninvasive and efficient new path for large‐scale early screening of lung cancer.

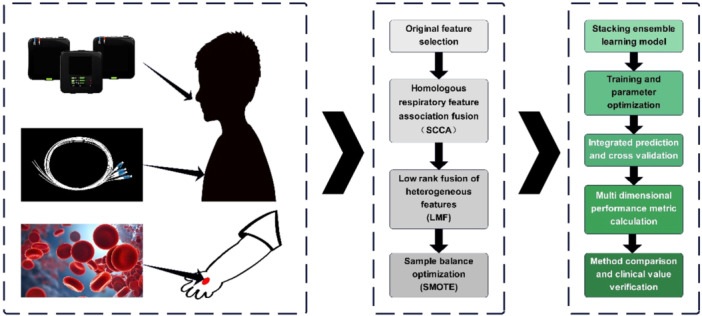

**Abbreviations**
ACC: accuracy
AI: artificial intelligence
AUC: area under the curve
CCA: canonical correlation analysis
CT: computed tomography
DCT: discrete cosine transform
F1_S: F1 score
HSF: high‐level statistical function
LLD: low‐level descriptor
LMF: low‐order multimodal fusion
MCC: Matthews correlation coefficient
MFCC: Mel‐scale frequency cepstral coefficient
PERS: precision
SCCA: Sparse canonical correlation analysis
SENS: sensitivity
SPEC: specificity
SPL: sound pressure level
STE: short‐term energy
TFN: tensor fusion network
ZCR: zero crossing rate

## 1 Background

Despite significant progress in areas such as the economy, technology, culture, and healthcare, modern medicine still faces challenges in combating malignant tumors, which remain one of the deadliest noncommunicable diseases in the world. Malignant tumors have complex pathogeneses, rapid development, and extremely high mortality rates. The diversity and complexity of this disease make preventing and treating lung cancer challenging. Lung cancer has become a significant threat to public health worldwide due to its unique biological characteristics and widespread epidemiological impact. Lung cancer was the most commonly diagnosed cancer in 2022, accounting for nearly 2.5 million new cases and one‐eighth of all cancers worldwide (12.4% of all cancers worldwide) [1]. The situation in our country is equally severe, with 820,000 new cases and 710,000 deaths in the same year (accounting for 17.9% of the new global cases), both ranking first in the world. Research shows that 75% of lung cancer patients in China are diagnosed at advanced stages, with a 5‐year survival rate of less than 15%. Currently, X‐rays and computed tomography (CT) are the mainstream methods for diagnosing lung cancer. X‐rays are widely used due to their low cost and convenient operation; however, the limitations of imaging quality and high risk of missed diagnoses are significant shortcomings that cannot be ignored. CT scans provide clearer images and can more accurately detect and locate lung nodules compared to X‐rays. However, complex imaging analysis may lead to confusion between lung cancer and other lung diseases, increasing the risk of misdiagnosis [2]. Early CT images require professional manual analysis, especially for the diagnosis of small and isolated pulmonary nodules [3], necessitating highly skilled doctors to conduct accurate evaluations and apply judgment [4]. Early screening for lung cancer also faces the potential risk of overdiagnosis when relying on imaging examinations. Research has shown that the CT radiation dose is enormous, and for every 100 mGy increase in CT radiation dose, the risk of developing hematological malignancies increases by 96%. The results of most individual studies [5, 6, 7, 8] and recent meta‐analyses have shown that repeated CT scanning increases the risk of leukemia, and even at low doses (10–15 mGy), the overall cancer risk still increases significantly [9], causing patients to lose the benefits gained from early diagnosis. In addition, the application and popularization of imaging technology are constrained by the allocation of medical resources and the level of regional economic development. The accessibility to imaging examinations is limited in resource‐constrained or economically underdeveloped regions, which, in turn, significantly impacts the coverage and effectiveness of early lung cancer screening and diagnosis.

The biomarker method mainly utilizes liquid biopsy technology for pathological detection, and research on biomarkers in the field of diagnosis is rapidly developing. For example, lactate dehydrogenase (LDH) [7, 10], C‐reactive protein (CRP) [11], and interleukin (IL)‐17 [12] have good predictive value for refractory *Mycoplasma pneumoniae* pneumonia (RMPP). Biomarkers, such as circulating tumor cells (CTCs) [8] and circulating tumor DNA (ctDNA) [13], provide real‐time biological information for assessing the risk of lung cancer in patients, which is of great significance for guiding personalized treatment and efficacy evaluations. However, these still face challenges and shortcomings in the diagnosis of lung cancer. First, although many specific biomarkers have been identified, the sensitivity and specificity of using biomarkers for detection need to be improved. Second, due to different individual conditions, especially the heterogeneity of tumors in cancer, identifying a widely applicable biomarker is a complex endeavor [14]. In addition, the high cost of this technology and the fact that most methods are still in the experimental stage are also reasons why they have not yet become a routine screening tool for lung diseases. In contrast, routine hematological testing has the advantages of non‐invasiveness, ease of repeated sampling, and low cost, making it an important supplement to imaging and liquid biopsy techniques. Wu et al. [15] used routine blood data to analyze the incidence characteristics of lung cancer disease, and ultimately selected 19‐dimensional blood data using feature extraction methods. The authors used random forest modeling to achieve a model accuracy of 94.74%. In 2022, Zhang et al. [16] used the SMOTE data balancing method combined with a random forest classification algorithm to identify lung cancer using recurrent microRNA, achieving an area under the curve (AUC) of up to 0.99. Unlike imaging, hematological testing is not affected by radiation and can detect disease signs before symptoms appear, which helps achieve early prediction. Hematological testing has a lower technical threshold than liquid biopsies, faster result acquisition speed, and is more suitable for large‐scale screening and real‐time monitoring. In addition, hematological testing can reflect biological information on tumors throughout the body, reflecting the heterogeneity and dynamic changes in tumors. However, this technology is in the research stage, and analyses of the correlation, specificity, and sensitivity between blood indicators and lung cancer are not sufficiently comprehensive.

With continuous advancements in sensor technology and the development of communication technology, the characteristics of small size, light weight, long battery life, and easy wearing of audio sensors enable them to flexibly collect medically related data in daily life [17]. Researchers have also accumulated results using artificial intelligence (AI) algorithms to diagnose and classify diseases using audio data [18]. In terms of lung diseases, Kosasih et al. [19] extracted wavelet features from cough sounds and other features to classify the diagnoses of pneumonia patients, with a sensitivity and specificity of 94% and 88%, respectively. Manshouri et al. [5] extracted features such as Mel frequency cepstral coefficients (MFCCs) from cough sounds and used a support vector machine (SVM) algorithm to detect and classify COVID‐19, with an accuracy of 95.86%. Fiber‐optic sensors have great potential for data acquisition and auxiliary evaluation in the medical field due to their advantages of anti‐electromagnetic interference, low loss, and high sensitivity. Chandana et al. [6] observed and monitored the respiratory rate of subjects by installing a fiber Bragg grating on the valve of a respiratory mask. In terms of home monitoring, Han et al. [20] embedded plastic optical fiber (POF) pressure sensors in mattresses to measure subjects' breathing and heart rates at different positions and postures to monitor sleep performance. In terms of wearable devices, Wang et al. [21] integrated high‐sensitivity D‐shaped POF sensors into elastic band structures, allowing for the monitoring of respiratory rates in different motion and physiological states of the human body.

In a study by Hu et al. [22], 1474 (46.0%) of 3203 patients who underwent surgery for primary lung cancer were admitted due to initial respiratory symptoms. Although chest tightness or difficulty breathing were relatively rare, cough and sputum were the two main complaints. It is worth noting that cancer risk can be predicted in a considerable number of early‐stage cancer patients based on their respiratory system condition. The fiber‐optic vibration signals collected by fiber‐optic sensors can reflect the fluctuating vibrations of the lungs during breathing, providing dynamic characteristics of the patient's breathing. The respiratory audio signals collected by audio sensors can reflect airway conditions and a portion of the lung tissue status. Although, as medical acquisition devices, fiber‐optic sensors are highly suitable for assisting in the collection of respiratory signal data in the medical field due to their anti‐electromagnetic interference, low loss, and high sensitivity, they are susceptible to external interference and unexpected patient movements. The characteristics of small size, light weight, long battery life, and easy wearing of audio sensors enable them to flexibly collect medically related respiratory audio data in daily life. However, the interference of environmental sound and human voice makes it difficult to ensure the robustness of their data.

Considering the limitations of existing imaging techniques, biomarker detection, and hematological testing methods in early cancer diagnosis, this study aims to establish a rapid, noninvasive, and low‐cost cancer prediction and evaluation system. Patient respiratory status data were collected using audio sensors and fiber‐optic sensors, and combined with routine blood detection indicators to analyze the changes in respiratory signals and hematological indicators in cancer patients and realize cross‐scale feature correlation between macroscopic respiratory physiological signals and microscopic blood indicators. Overall, combining the effectiveness of noninvasive blood routine screening, the portability of audio detection, and the high accuracy of fiber‐optic diagnosis, feature engineering was used to extract modal features, and the SCCA‐LMF fusion method was used to optimize multimodal fusion information and mine key features from multiple perspectives. An improved stacking ensemble learning model was constructed in an attempt to break through the traditional dependence of lung cancer diagnosis on a single technical path, and through the joint modeling of macroscopic respiration and microscopic blood dimensions, it improved the efficiency and comprehensiveness of a clinical diagnosis of lung cancer, opening up a new methodological path for cancer diagnosis.

## 2 Methods

### 2.1 Sample Data Source

The data in this article were sourced from the Second Hospital of Lanzhou University (2022–2024). A total of 360 samples were included, including patients with lung cancer, chronic obstructive pulmonary disease, tuberculosis, and normal individuals. The classification of all samples was based on standardized diagnostic reports obtained after an immediate clinical diagnosis. The age of patients clinically diagnosed with lung cancer and those diagnosed with nonlung cancer was significantly different. The average age of the lung cancer patients was significantly higher than that of the non‐lung cancer patients (60.0 ± 11.7 vs. 48.3 ± 18.7) (independent sample *t*‐test *p* < 0.001). In terms of gender, the number of male cancer patients was significantly higher than that of female patients (69.9% vs. 30.1%) (chi‐squared Fisher's exact test (two‐sided) *p* = 0.007). Due to insufficient clinical statistics, the patient's smoking history is only presented as baseline data here. Detailed statistical information is shown in Table 1.

**Table 1:** Summary demographics.

Design	All subjects (*n* = 360)	Subjects with lung cancer (*n* = 282)	Subjects without lung cancer (*n* = 78)	*p*‐value
Age (years)				
Mean ± SD	57.5 ± 14.3	60.0 ± 11.7	48.3 ± 18.7	*p* < 0.001
Range (min to max)	16.0–87.0	26.0–87.0	16.0–81.0	/
Median (Q1, Q3)	59.0 (49.0, 68.0)	60.0 (53.0, 68.0)	50.0 (31.0, 64.0)	/
Sex *n* (%)				*p* = 0.007
Male	238 (66.1)	197 (69.9)	41 (52.6)	/
Female	122 (33.9)	85 (30.1)	37 (47.4)	/
Smoker (incomplete statistics)	63 (17.5)	49 (17.4)	14 (17.9)	/

Each sample included the respiratory fiber vibration mode, the respiratory audio sound mode, and the hematological routine index mode. Out of 360 samples, 282 were clinically diagnosed with lung cancer and labeled as “1” (positive). The remaining 78 cases were clinically diagnosed as nonlung cancer cases and recorded as “0” (negative). The Ethics Committee of the Second Hospital of Lanzhou University approved this study (Ethics approval number: 2022a‐569). All participants signed an informed consent form to participate in the study after listening to an oral explanation of the study.

### 2.2 Respiratory Signal Modality

**Figure 1:**
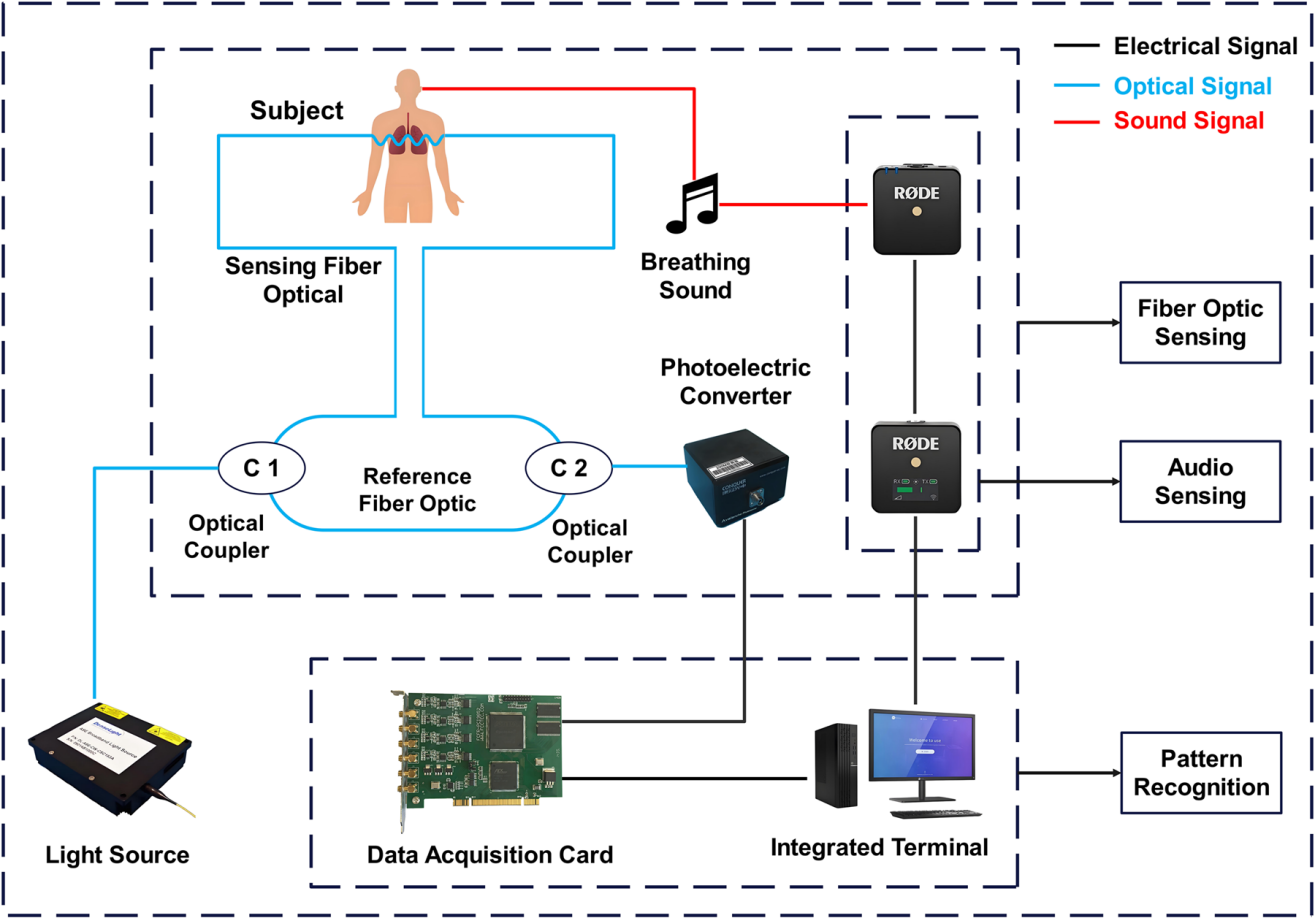
Schematic diagram of respiratory signal modal acquisition. Key equipment parameter description: Light source: Output power > 10 mW, center wavelength 1550 nm t 2 nm, edge mode suppression ratio > 45 dB, single‐mode fiber FC/Apc interface, 15s0nm single‐modtail fiber; Photoelectric converter: $pectral response range of 850–1650 nm, response freguency can reach 200 MHz; data acquisition card: Trigger sampling rate of 100 M$/s/CH, continuous real‐time transmission of 70MBytes/S; audio sensing: Frequency range: 50 Hz–20 kH > maximum propagation range: 200 m.

**Figure 2:**
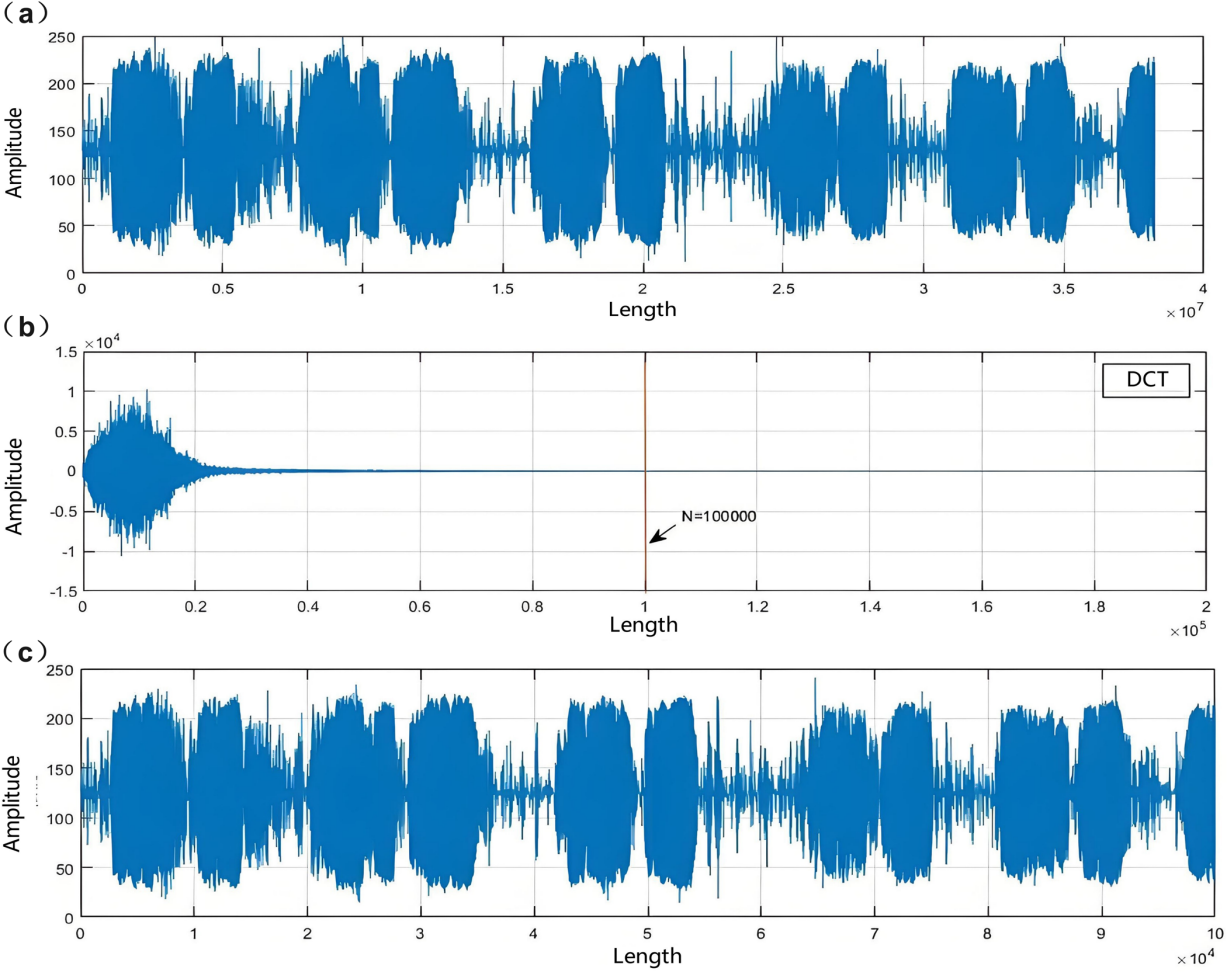
Raw signal denoising processing. (a) Original respiratory signal. (b) DCT coefficients. (c) Reconstructed signal.

Lung cancer patients often experience symptoms such as abnormal lung sounds, difficulty breathing, and shortness of breath. This study used both sensing fibers and audio collectors to collect patients' respiratory waveforms and lung sound signals. The data collection process is shown in Figure 1. In this study, the sensing fiber was placed between the first and second ribs of the patient to collect their respiratory vibration signals, with a fixed collection time of around 20 s. Background interference includes human language, footsteps, periodic tones from medical devices, and various other interferences from patient movements. In the process of fiber‐optic signal acquisition, the first step is to compress and preprocess the acquired data to reduce computational complexity. Due to significant interference caused by electromagnetic interference and unexpected body movements of the equipment, the study applied the discrete cosine transform (DCT) to the original respiratory signal to obtain the corresponding coefficients (Figure 2). During this process, signal energy is concentrated at low frequencies, and high frequencies tend to zero. Therefore, high‐frequency noise was filtered out with a threshold of 100,000, and the low‐frequency part was subjected to DCT inverse transform to obtain a reconstructed low‐frequency physiological signal.

This study used methods such as wavelet transform and Fourier transform to extract features of the fiber‐optic signal mode, including time‐domain features, frequency‐domain features, and wavelet transform features, totaling 79 dimensions. The time‐domain features had a total of 12 dimensions. The frequency domain features had a total of 39 dimensions. The wavelet transform features had a total of 28 dimensions.

Considering the acquisition method and actual situation of the pathological audio signals, this study used a wireless microphone (RODE Wireless GO II, Beijing, China, with an omnidirectional recording direction, 16 bits/sample, and a 48 kHz sampling rate) as the acquisition device. During collection, the microphone was worn under the patient's collar, aimed at the patient's mouth and nose at a distance of 3–5 cm to collect the patient's respiratory audio signals, with an average recording length of 20 s.

In the process of audio collection, pure respiratory audio signals were obtained by denoising the audio signals by collecting them in relatively quiet environments to reduce interference. Subsequently, using the calculation results of short‐term energy, short‐term zero crossing rate, and so on, the audio signal endpoints were detected using the double‐threshold method, and the effective parts were cut and re‐combined to obtain effective signals that were conducive to algorithm analysis.

The feature extraction in the respiratory audio signal modal includes low‐level descriptors (LLDs), calculated on an audio frame to represent its features. High‐level statistical functions (HSFs) are features obtained through statistical analysis based on LLDs, such as mean and maximum values. The audio signal features extracted in this study can be roughly divided into three categories: frequency domain features, sound quality features, and rhythm features, totaling 523 dimensions. The frequency domain features included the HSF features of the 39‐dimensional Mel‐scale frequency cepstral coefficients (MFCCs), totaling 273 dimensions. The sound quality characteristics included the HSF characteristic of the resonance peak center frequency and its corresponding bandwidth, as well as the HSF characteristics of signal frequency and amplitude perturbation, totaling 130 dimensions. The phonetic features included fundamental frequency, energy, and the HSF zero crossing rate, totaling 120 dimensions. Considering the practical needs, only 100‐dimensional high‐contributing audio signal‐modality features, including LLDs and HSFs, were selected in the subsequent modal fusion process (details on the 79‐dimensional fiber‐optic vibration mode and 523‐dimensional respiratory audio mode appear in the supplementary document).

**Figure 3:**
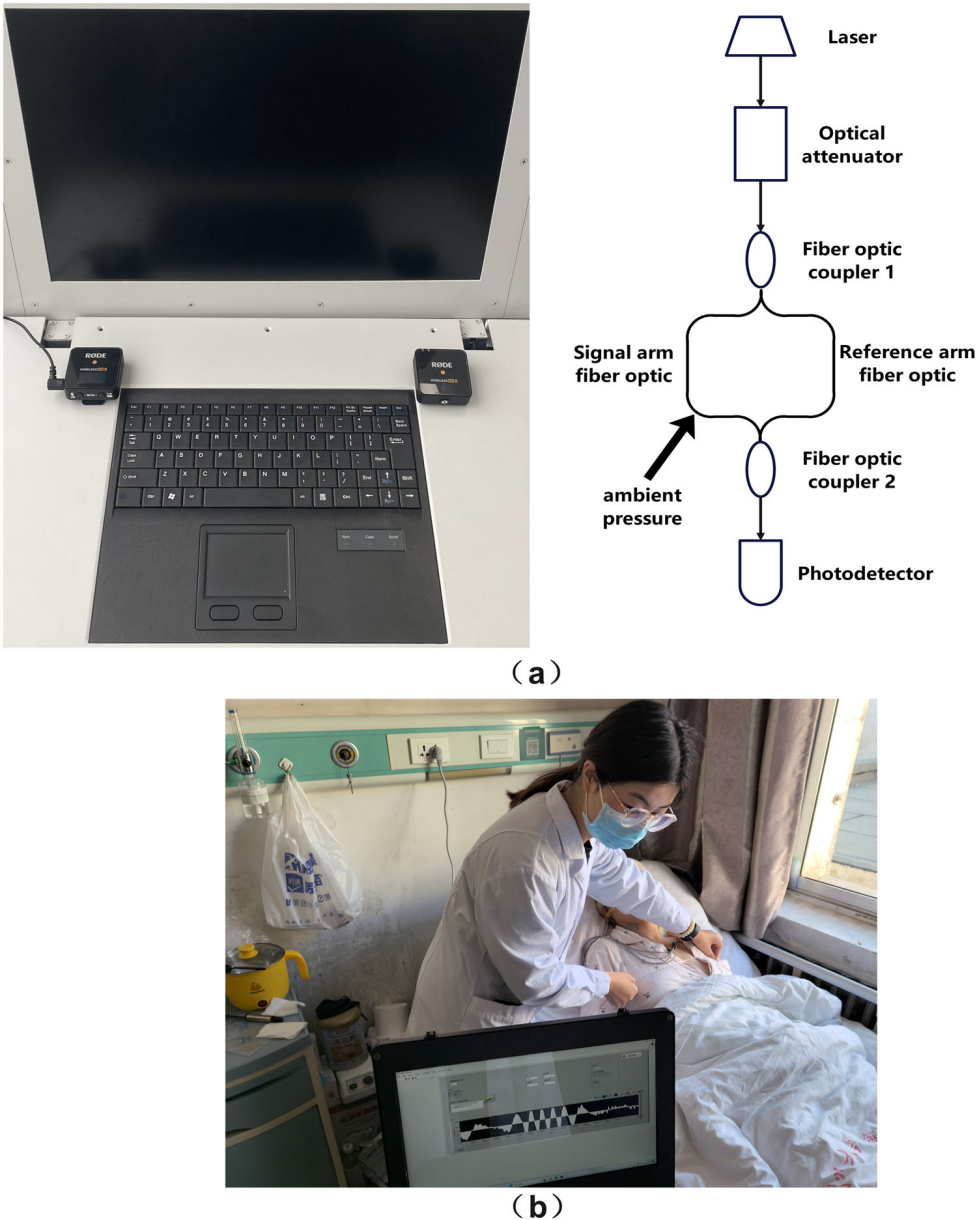
Respiratory signal modality. (a) Diagram of respiratory signal modality acquisition equipment. (b) Schematic diagram of respiratory signal modal acquisition scenario.

In this study, the obtained sensing fiber vibration modes and respiratory audio modes are collectively referred to as respiratory signal modes. The specific acquisition process diagram and equipment physical images of the respiratory signal modes are shown in Figures 1 and 3.

### 2.3 Blood Index Modality

**Figure 4:**
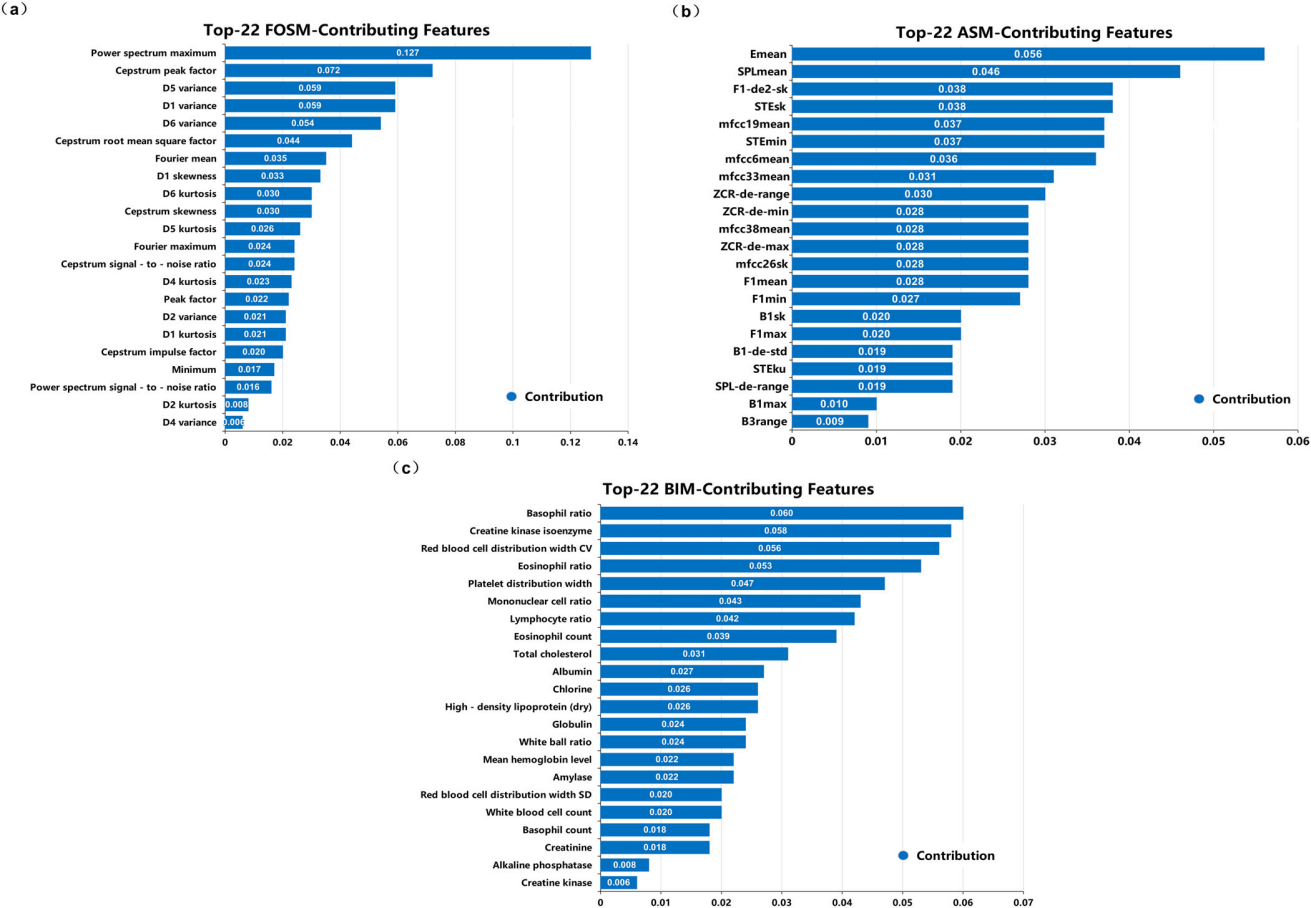
Ranking diagram of the contribution of the top 20 optical fiber modes. (a) Fiber optic mode. (b) Audio modality. (c) Blood modality.

The blood index modal data and respiratory signal modal used in this article are both homologous data and were collected from the Second Hospital of Lanzhou University. A total of 752 samples were collected for the experiment. After data screening and difference supplementation, 360 samples, including lung cancer, chronic obstructive pulmonary disease, tuberculosis, and normal samples, were included for model training and testing. All sample data were the final predicted results provided by professional doctors based on clinical imaging diagnosis and liquid biopsy reports. The data included a total of 56 hematological indicators, as shown in Table 2. Based on the impact of features on the classification performance of the model, we evaluated the contribution of each feature in distinguishing between lung cancer and nonlung cancer samples. We analyzed the correlation between features and target variables, the importance score in the model, and the contribution to differences in data distribution. We also sorted the classification values of all features from high to low. As shown in Figure 4, we ranked the top 22 features of the three modalities in descending order of contribution. We found that the contribution values of the top 20 features were significantly higher than those of the other features. Therefore, in the feature screening stage, we uniformly selected the top 20 features with the most prominent contribution from the three modalities and ranked them.

**Table 2:** Summary table of 56 hematology indicators.

Fifty‐six hematological index parameters
Albumin, white blood cell ratio, white blood cell count, alanine aminotransferase, large platelet ratio, monocyte ratio, monocyte count, low‐density lipoprotein (dry), amylase, carbon dioxide binding rate, calcium, triglycerides, high‐density lipoprotein (dry), glutamyl transferase, red blood cell distribution width CV, red blood cell distribution width SD, red blood cell count, average red blood cell volume, hematocrit, creatinine, creatine kinase, creatine kinase isoenzyme, potassium, indirect bilirubin (dry), alkaline phosphatase, lymphocyte ratio, lymphocyte count, phosphorus, chlorine, magnesium, sodium, urea, urea/creatinine, uric acid, average hemoglobin content, average hemoglobin concentration, average platelet volume, glucose, globulin, lactate dehydrogenase, eosinophil ratio, eosinophil count eosinophil ratio, eosinophil count, aspartate/alanine, aspartate aminotransferase, hemoglobin, platelets, platelet distribution width, platelet hematocrit, direct bilirubin (dry), neutrophil ratio, neutrophil count, total cholesterol, and total bilirubin

### 2.4 Sparse Canonical Correlation Analysis Feature Fusion

Although the frequency characteristics in the fiber‐optic vibration mode are similar to the rhythmic characteristics in the respiratory audio mode, they exhibit different structural features in the one‐dimensional representation of the respiratory signal. Therefore, the key to this study was how to extract effective information from these two features and fuse them. SCCA) [23] is an extended version of CCA [24], aimed at simplifying models and improving interpretability by introducing sparsity constraints. Traditional CCA aims to reveal the maximum correlation between two multidimensional variable sets by finding a linear combination between them. However, CCA may be affected by redundant or irrelevant variables, making the model complex and difficult to explain.

SCCA addresses this issue by adding sparse constraints on top of CCA to optimize the model's simplicity and interpretability. SCCA achieves effective feature selection by introducing L1 regularization (LASSO) to make the coefficients of certain features zero. This method not only simplifies the model structure but also improves computational efficiency.

In this study, the frequency characteristics (F‐frequency: $F_{\text{Fr}}$) of the selected fiber‐optic vibration mode and the rhythmic characteristics (A‐rhythm: $A_{\text{Rh}}$) of the respiratory audio mode were fused through SCCA to obtain the clinical features of lung cancer. It can be mathematically expressed as in the below equation (Sparse canonical correlation analysis formula):

(1) $$\max_{a,b}\frac{a^{T}{F_{\text{Fr}}}^{T}A_{\text{Rh}}b}{\sqrt{\left(a^{T}{F_{\text{Fr}}}^{T}F_{\text{Fr}}a\right)\left(b^{T}{A_{\text{Rh}}}^{T}A_{\text{Rh}}b\right)}}-\lambda_{1}{\left\|a\right\|}_{1}-\lambda_{2}{\left\|b\right\|}_{1}.$$

First, the linear correlation between the fiber frequency characteristics and the vocal characteristics under the projections of *a* and *b* was calculated using $a^{T}{F_{\text{Fr}}}^{T}A_{\text{Rh}}b$. Then, the norms of vectors *a* and *b* in the fiber frequency characteristics and vocal characteristics were calculated using ${(a}^{T}{F_{\text{Fr}}}^{T}F_{\text{Fr}}a)(b^{T}{A_{\text{Rh}}}^{T}A_{\text{Rh}}b)$, respectively, to normalize them and ensure that the correlation between the two sets of features was measured rather than the length of the vectors. $\lambda_{1}{\left\|a\right\|}_{1}$and$\lambda_{2}{\left\|b\right\|}_{1}$use the strength of the regularization term of the L1 norm of the two regularized sparse control vectors a and b, and the nonzero coefficients in the L1 norm penalty vector to make some sparsity zero, achieving the purpose of feature selection.

In summary, first, the SCCA method was used to select the appropriate vectors a and b to maximize the correlation between fiber frequency features and acoustic features, while L1 regularization was used to eliminate unimportant feature coefficients, achieving feature selection for both sets of features. The SCCA method can find a low‐dimensional space with two sets of features in common, reduce noise interference, highlight key respiratory mode information, identify effective respiratory mode information more accurately, and enhance the interpretability of the model.

### 2.5 Low‐Rank Multimodal Feature Fusion

It is crucial to integrate the frequency characteristics of the fiber‐optic vibration mode and the rhythmic characteristics of the respiratory audio mode through SCCA, and then combine the fusion product with the blood index mode of lung cancer for comprehensive signal analysis. Liu et al.'s low‐order multimodal fusion (LMF) method [25] achieved multimodal fusion through parallel tensor and weight decomposition, using low‐order factors for modality‐specific analysis, solving the computational efficiency problem in tensor fusion networks (TFNs) [26]. In terms of performance comparison, the computational speed of LMF was more than twice that of TFN, and its complexity was reduced from exponential to linear level through low‐rank decomposition, with a significant reduction in the number of parameters. Experiments using both methods showed that LMF outperformed TFN while maintaining performance. For example, using the CMU‐MOSI data set for multimodal sentiment analysis, LMF's MAE value is 0.912, which is better than TFN's 0.970. When the POM data set was used for trait analysis, the MAE value of LMF was 0.796, which was better than TFN's 0.886. LMF has greater flexibility and can better adapt to different modalities and tasks. This study used SCCA to fuse the frequency features in the fiber‐optic vibration mode with the rhythmic features in the respiratory audio features to generate the clinical features of lung cancer (CFLC). Whiood cell‐related features in the blood index mode were used as laboratory markers of lung cancer (LMLC). The two were fused through LMF to obtain optimal multimodal fusion features for lung cancer, denoted as optimal fusion features for lung cancer. The goal was to effectively integrate homologous and heterologous lung cancer feature information, thereby utilizing data complementarity for accurate prediction.

**Figure 5:**
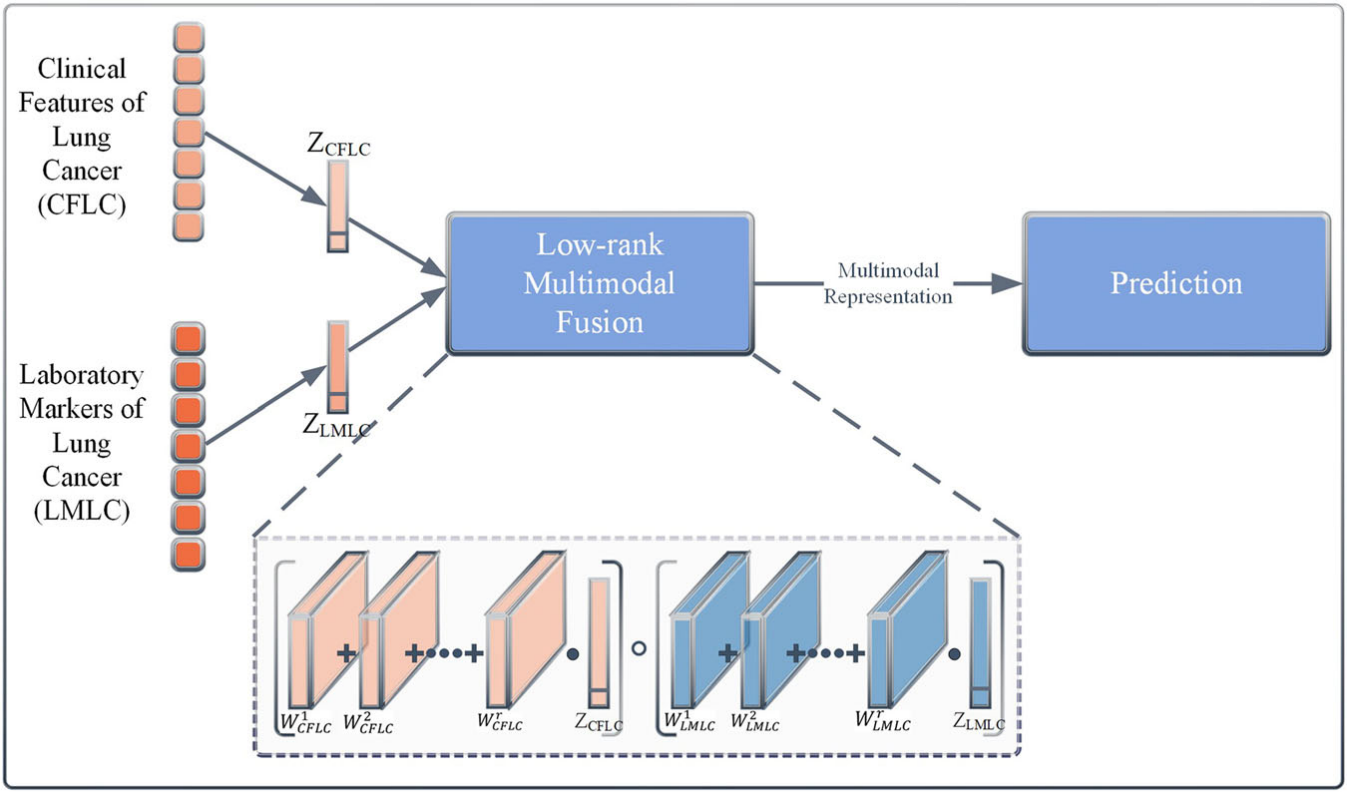
Low‐rank multimodal fusion method.

In this study, CFLC and LMLC feature vectors underwent modal fusion. The LMF method used is shown in Figure 5. First, bias terms were added to the feature matrices of each modality to obtain bias matrices *Z*_CFLC_ and *Z*_LMLC_, so that the input of each modality could adapt to the low‐rank matrix factorization method. Afterward, the weight tensor W was subjected to low‐rank decomposition to obtain the weight low rank specific factors $W_{\mathrm{CFLC}}^{r}$ and $W_{\mathrm{LMLC}}^{r}$. The feature fusion was transformed from the weighted combination of the weight matrix *W* and the outer product of the feature tensors *Z*_CFLC_ and *Z*_LMLC_ into a series of element products of the weight low rank specific factors $W_{\mathrm{CFLC}}^{r}$, $W_{\mathrm{LMLC}}^{r}$, and the feature tensors *Z*_CFLC_ and *Z*_LMLC_, thereby generating the final output fusion vector ℎ. LMF not only captures the interaction information between different modalities but also achieves the parallel decomposition of feature representation *Z* and weight tensor *W*, greatly reducing computational complexity.

**Figure 6:**
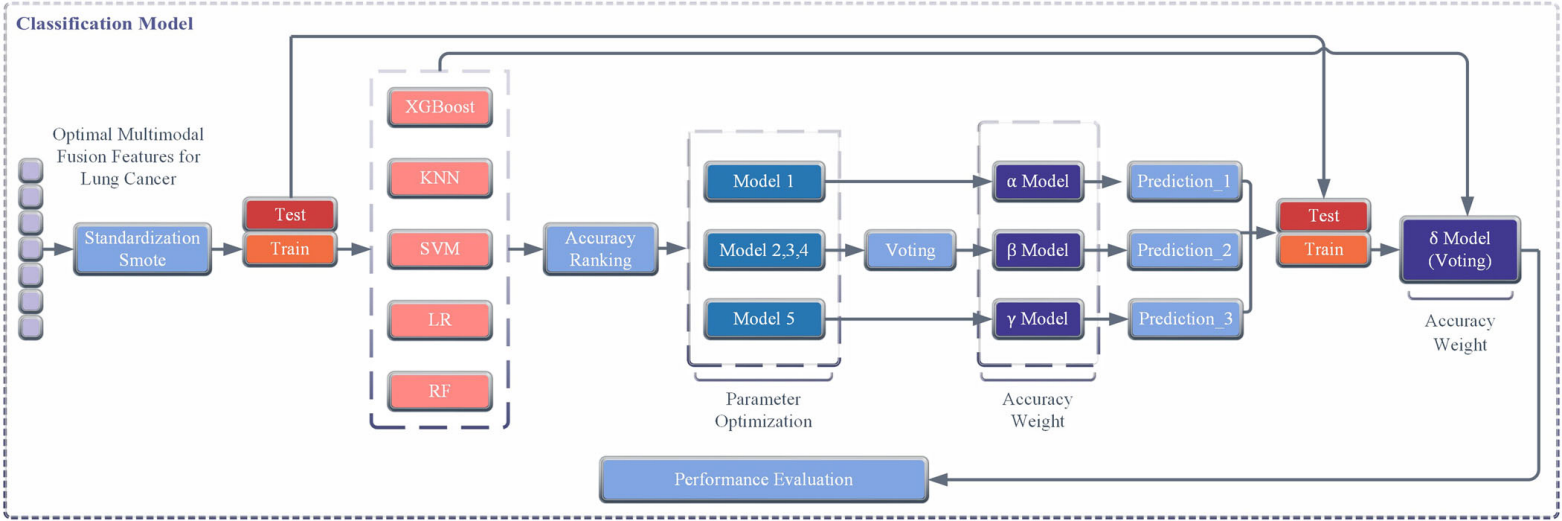
Flowchart of the classification model.

Classification model: In terms of the classification processing of the fused data, this study established a smote‐voting‐stacking (SVS) ensemble learning model that integrates different machine‐learning algorithms for data classification, as shown in Figure 6. First, the Smote algorithm was used to oversample the data to address the imbalance between the lung cancer and nonlung cancer samples, thereby reducing the risk of overfitting. The model prototype adopted a stacking ensemble learning algorithm, which integrates five basic classifiers, including SVM, XGBoost, and so on, and allocates decision weights based on the accuracy of each classification to improve predictive performance. During the process, a grid search method was used for parameter optimization to ensure the coverage of all possible parameter configurations, avoid missing potential optimal solutions, and stably identify the optimal global parameters to further improve the final performance. The model was continuously trained on the training data through cross‐validation, and its performance was repeatedly measured using accuracy, specificity, sensitivity, and F1 scores. Then, based on the comparative evaluation results of all parameter combinations, the parameter combination with the best evaluation index was selected as the final parameter of the model, thus completing the optimization process of the integrated model parameters. In the process of model training, this study adopted a five‐fold cross‐validation method: the training set was further divided into five equal parts to avoid overfitting, with four parts used to train the model each time and the remaining one for testing. This cycle was repeated five times, and the results of each test were different. Finally, the average of the five results was taken as the final evaluation of the model's performance. This method allows the model to be tested on different subsets of data, ensuring its stable performance in real‐world scenarios. In addition, when selecting key features, such as fiber‐optic vibration signals, respiratory audio, and effective parameters in blood indicators, the study used 10‐fold cross‐validation to screen each type of feature separately. By testing different numbers of feature combinations multiple times, the subset of features that can achieve the highest accuracy of the model was found, avoiding the introduction of irrelevant or redundant information to interfere with the judgment. Ultimately, the hybrid ensemble learning model showed good generalization ability and could be extended to other data and modal fusion scenarios.

## 3 Results

### 3.1 Experimental Prediction Criteria

The experiment used accuracy, precision, sensitivity, specificity, F1 scores, ROC curves, and AUC value indicators to verify the effectiveness of the proposed model [26, 27], where accuracy was defined as in Equation (2). ROC is also known as the receiver operating characteristic curve. The horizontal axis represents the false positive rate (FPR) for determining pneumonia as nonpneumonia, while the vertical axis represents the true positive rate (TPR) for correctly determining pneumonia [28]. After exploring all the thresholds in the experiment, the threshold corresponding to the maximum Youden index point was selected as the final result for plotting the ROC curve. The advantage of using a single threshold is that it can meet clinical needs well, is easy to use, and the results are intuitive and easy to understand. In clinical practice, it is usually necessary to follow a fixed diagnostic threshold. Focusing on a single threshold can directly evaluate the rates of missed and misdiagnosed cases, avoiding the complexity of multi‐threshold analysis and helping healthcare professionals quickly understand and apply it to screening decisions. These advantages were validated in the work of Hassanzad et al., in which the optimal cut‐off point for the ROC curve of biomarkers was determined [29]. At the same time, it reduces the consumption of computing resources, improves screening efficiency, provides unique and stable results, promotes the standardized comparison of different batches of screening data or different testing methods, and ensures diagnostic consistency (see multithreshold curve reference supplementary document). As defined in Equations (3) and (4).

(2) $$\mathrm{Accuracy}=\frac{\mathrm{TP}+\mathrm{TN}}{\mathrm{TP}+\mathrm{FP}+\mathrm{TN}+\mathrm{FN}},$$

(3) $$\mathrm{FPR}=\frac{\mathrm{FP}}{\mathrm{TN}+\mathrm{FP}},$$

(4) $$\mathrm{TPR}=\frac{\mathrm{TP}}{\mathrm{TP}+\mathrm{FN}}.$$

The better the performance of the classification model, the larger the TPR and the smaller the FPR, and the closer the ROC curve is to the upper left. Since it is difficult to judge the quality of a model solely by observing the ROC curve, the area under the ROC curve (AUC) is introduced, which represents the area enclosed by the ROC curve and the *X*‐axis. If the AUC value of a model is equal to 0.5, it indicates that the model is invalid. The closer the AUC is to 1, the more reliable the classification model's performance.

Matthews correlation coefficient (MCC) is a statistical measure used to evaluate the strength of a linear relationship between two variables, which is a special case of the PHI coefficient [30]. In binary classification problems, MCC provides a single numerical value to comprehensively evaluate the performance of classification models by considering the four basic components of the confusion matrix: true examples (TN), true negative examples (TP), false negative examples (FN), and false positive examples (FP). Thus, this indicator is widely used in the model evaluation of imbalanced data and will only give a high score when the performance of all four confusion matrix categories is good. When the predictive results of the model are completely accurate, the value of MCC is “1.” If the predictive results of the model are completely incorrect, the value of MCC is −1. An MCC value of “0” indicates that the performance of the model is not different from random guessing. MCC was calculated as in Equation (5) below.

(5) $$\mathrm{MCC}=\frac{\mathrm{TP}\times \mathrm{TN}-\mathrm{FP}\times \mathrm{FN}}{\sqrt{\left(\mathrm{FP}+\mathrm{TP})(\mathrm{TP}+\mathrm{FN})(\mathrm{TN}+\mathrm{FP})(\mathrm{TN}+\mathrm{FN}\right)}}.$$

### 3.2 Experimental Results and Analysis

The experiment used the Bayesian parameter optimization method to identify the hyperparameter combination that achieved the optimal model performance. Five‐fold cross‐validation was used to divide the data set into five subsets, each with the same sample category distribution as the overall sample category, to evaluate the generalization effect of the proposed model. One subset was selected as the test set and the rest as the training set each time. The experiment first evaluated the performance of single‐modality classification, then evaluated the effect of feature selection, and finally, the classification effect of multimodal fusion of the selected features. All evaluation models used the proposed hybrid ensemble learning classification model.

#### 3.2.1 Single‐Modality Classification Performance Evaluation

**Figure 7:**
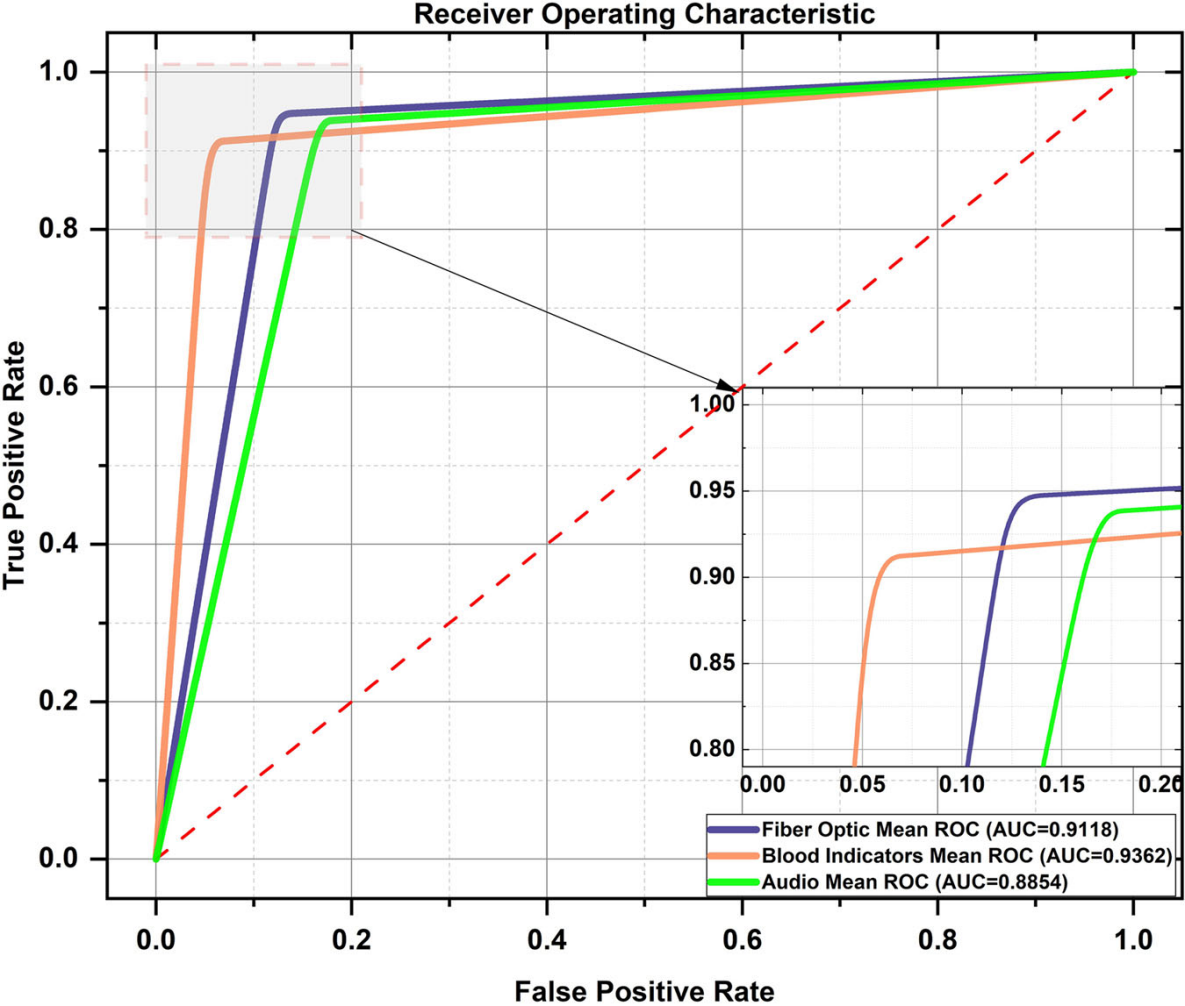
ROC curves for the three models of lung cancer classification.

The experiment first evaluated the classification performance of three single‐modality data modes: the fiber‐optic vibration mode, the respiratory audio mode, and the blood index mode. Then, the classification performance of the three single‐modality data representative features after feature selection is evaluated. Finally, the classification performance of multimodal global feature fusion was evaluated. Table 3 shows the detailed experimental results of single‐modality data in the classification model, and Figure 7 shows the classification ROC curves of three types of single‐modality data. We selected ROC curves under a single threshold when the AUC values of the three modalities reached their maximum to meet the high‐performance requirements for the rapid clinical prediction and discrimination of lung cancer.

**Table 3:** Evaluation metrics for single‐modality data.

Design	ACC	PERS	SENS	SPEC	F1_S	AUC	MCC
Fiber‐optic vibration modality	0.9115	0.8833	**0.9464**	0.8771	0.9137	0.9118	0.8251
Respiratory audio modality	0.9166	**0.9574**	0.9375	0.8333	**0.9473**	0.8854	0.7484
Blood index modality	**0.9363**	0.9340	0.9397	**0.9327**	0.9366	**0.9362**	**0.8733**

*Note:* The bolded data in the table represent the optimal performance of the three modalities under seven performance indicators.

The experimental results of two modality data showed that among the three single‐modality features, although the accuracy, specificity, AUC, and MCC values of the blood index features were higher than those of fiber‐optic vibration features and respiratory audio features, the sensitivity of the fiber‐optic vibration features was the best compared to the other two modalities. The accuracy and F1 scores of the respiratory audio features were the best compared to the other two modalities. Moreover, the comprehensive classification performance of the three single‐modality datasets was basically similar. Therefore, feature processing and feature fusion must be performed to effectively utilize the respective advantages of the three datasets.

#### 3.2.2 Performance Evaluation of Feature Selection Classification

**Figure 8:**
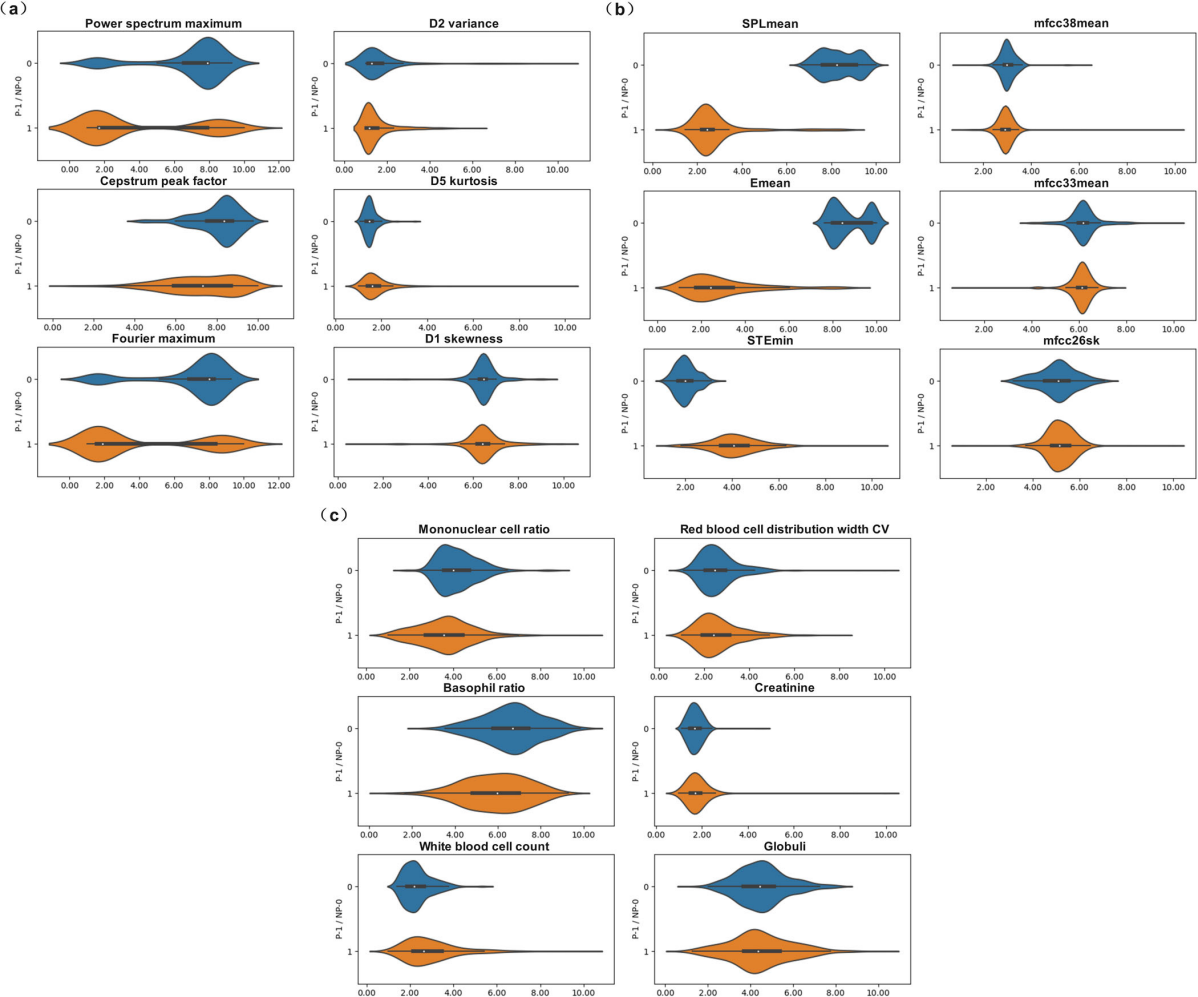
Comparison diagram of highly contributing features in the three modalities. (a) The top three fiber‐optic vibration modality contributing features (left) and the bottom three contributing features (right). (b) The top three respiratory audio modality (left) contributing features and the bottom three contributing features (right). (c) The top three blood index modality contributing features (left) and the bottom three contributing features (right).

This study adopted a processing flow of sorting‐classification‐optimization‐fusion when dealing with multimodal feature data. First, the contribution of the features of the three modalities was ranked, and the top 20 dimensional features were selected as the ranking results (Table 4). Then, violin plots were drawn for the top three and bottom three contributing characteristics in each modality (Figure 8) to observe their distribution in lung cancer and non‐lung cancer. The figure shows that although most of the high‐contributing features in the first 20 dimensions had significantly different data distributions, some feature distributions were not significantly different between lung cancer and non‐lung cancer. Therefore, further feature selection was still necessary.

**Table 4:** Summary of the top 20 dimensions of the three modalities.

**Twenty fiber‐optic vibration modal parameters:**
D5 variance, D2 variance, D1 variance, D6 variance, D6 kurtosis, D1 skewness, D1 kurtosis, D5 kurtosis, D4 kurtosis, power spectrum maximum, Cepstrum peak factor, Fourier maximum, Cepstrum skewness, Cepstrum root mean square factor, Cepstrum signal‐to‐noise ratio, power spectrum signal‐to‐noise ratio, Fourier mean, Cepstrum impulse factor, peak factor, and minimum
**Twenty respiratory audio modal parameters:**
SPLmean, Emean, ZCR‐de‐range, STEsk, ZCR‐de‐min, ZCR‐de‐max, STEmin, STEku, SPL‐de‐range, mfcc19mean, mfcc6mean, mfcc33mean, mfcc38mean, mfcc26sk, B1‐de‐std, B1sk, F1‐de2‐sk, F1mean, F1max, and F1min
**Twenty blood index modal parameters:**
Platelet distribution width, albumin, red blood cell distribution width CV, white cell ratio, mean hemoglobin level, red blood cell distribution width SD, mononuclear cell ratio, basophil ratio, eosinophil count, white blood cell count, eosinophil ratio, basophil count, lymphocyte ratio, creatine kinase isoenzyme, amylase, chlorine, total cholesterol, high‐density lipoprotein (dry), and creatinine

Abbreviations: B1, bandwidth at the resonance peak's center frequency; de, first‐order difference; de2, second‐order difference; E, logarithmic energy value; F1, center frequency of resonance peak; ku, kurtosis; max, maximum; min, minimum; sk, skewness; SPL, sound pressure level; std, standard deviation; STE, short‐term energy; ZCR, zero crossing rate.

**Figure 9:**
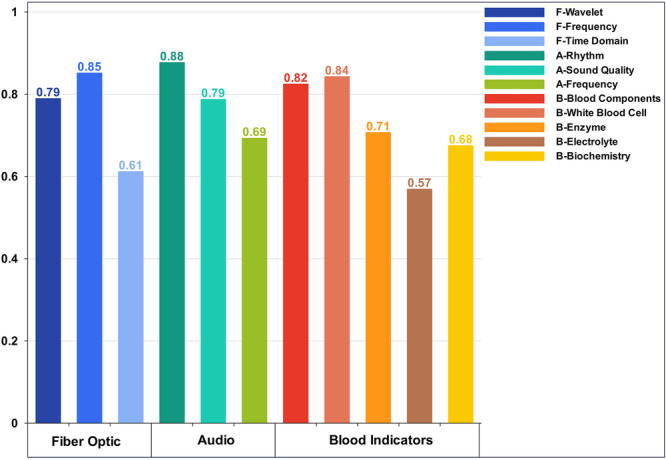
AUC histogram of different types of characteristics of the three modalities.

**Figure 10:**
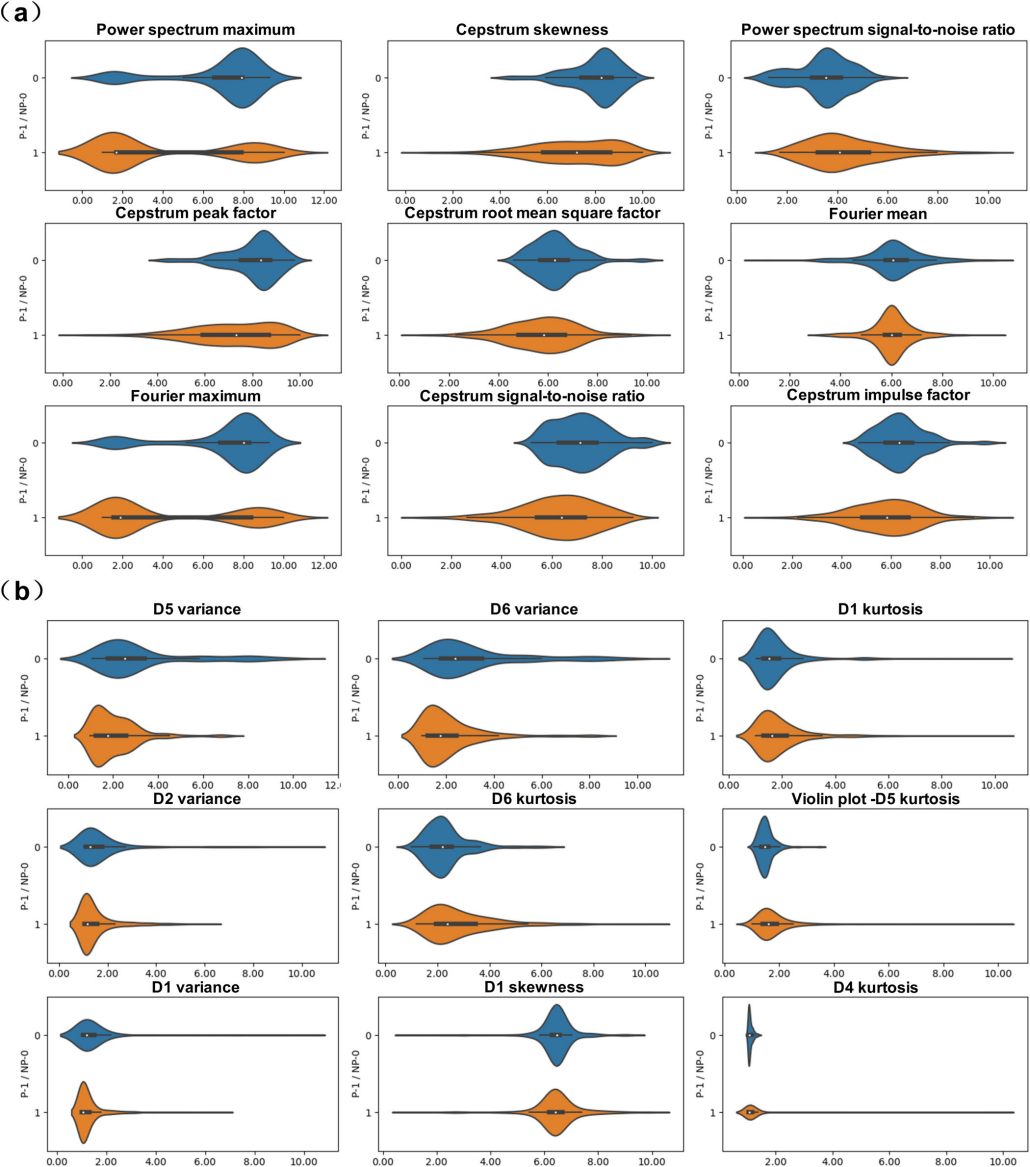
Violin plot of the fiber‐optic vibration modality. (a) Violin plot of the frequency features in the fiber‐optic vibration modality. (b) Violin plot of the wavelet domain features in the fiber‐optic vibration modality.

This study conducted cluster analysis on the top 20 dimensional contribution features of each modality based on the classification of the three modality data sources to further refine the features. Specifically, the fiber‐optic vibration mode was divided into three categories: the time domain, frequency domain, and wavelet domain. The respiratory audio mode was divided into three categories: the frequency domain, sound quality, and rhythm. The routine hematological indicators were divided into five categories: blood components, white blood cell‐related, enzyme‐related, electrolyte‐related, and biochemical indicator characteristics. In the first 20 optical fiber vibration modes, 9 dimensions came from the wavelet domain, 9 dimensions came from the frequency domain, and 2 dimensions came from the time domain. The wavelet domain features mainly reflect respiratory rhythm, abrupt changes, and specific points. The frequency domain features reveal the respiratory rate, stability, and nonlinear dynamic characteristics of the respiratory system. Time domain features reflect lung ventilation function, respiratory patterns, and respiratory resistance [31]. In the first 20 dimensions of respiratory audio modality, 9 dimensions were derived from prosodic features, 6 dimensions from acoustic features, and 5 dimensions from frequency features [32]. The frequency domain features reflect spectral envelope information on respiratory audio, the condition of the respiratory obstruction, and its morphology and size. The sound quality characteristics reveal the texture, timbre, and noise components of breathing sounds. The phonetic features mainly reflect the regularity of the respiratory cycle, the duration and intensity of breathing, and other information [33]. In the first 20 dimensions of routine hematological indicators, 7 dimensions came from blood components, 7 dimensions from white blood cell‐related features, 2 dimensions from related enzymes, 1 dimensions from electrolytes, and 3 dimensions came from biochemical indicators. The characteristics of blood components reflect the patient's blood oxygen‐carrying capacity and inflammatory response [34]. The characteristics related to white blood cells reflect the immune system status of the patients [35, 36, 37]. The enzymatic characteristics are related to specific organ functions and diseases. The electrolyte characteristics reveal the intracellular and extracellular environment, as well as neuromuscular function. Biochemical indicators reflect the functional status of multiple organ systems. Next, the classification performance of various features was evaluated. The experimental results are shown in Table 5. A bar chart comparing AUC values is shown in Figure 9. The classification performance of 9 dimensional eigenvalues using the fiber‐optic vibration mode frequency domain mode was higher than that of the other classes. The reason is that the statistical analysis found that among the features of the 9 dimensional wavelet domain modality, only the 3 dimensional features had significant differences in data distribution between lung cancer and nonlung cancer (Figure 10a). In the 9 dimensional frequency domain modality features, significant differences were found in the distribution of 8 dimensional features between lung cancer and nonlung cancer data (Figure 10b). Due to the limited dimensionality of the time‐domain features, they were not included in the preferred feature range. Therefore, this study selected the 9 dimensional characteristic values of the frequency domain of the fiber‐optic vibration modes as the preferred features. Using the same analysis method as for the fiber‐optic vibration mode, 9 dimensional features of rhythm and 7 dimensional features related to white blood cells were selected as the preferred features for the respiratory audio mode and the blood index mode, respectively.

**Table 5:** Evaluation metrics for different types of characteristics in the three modalities.

Design	ACC	PERS	SENS	SPEC	F1_S	AUC	MCC
F‐Wavelet	0.7897	0.8229	0.7493	0.8300	0.7804	0.7896	0.5860
F‐Frequency	**0.8516**	**0.8544**	**0.8553**	**0.8480**	**0.8507**	**0.8517**	**0.7105**
F‐Time domain	0.6111	0.6041	0.7323	0.4917	0.6518	0.6120	0.2319
A‐Rhythm	**0.8761**	**0.8954**	**0.9575**	**0.7964**	**0.9088**	**0.8770**	**0.7552**
A‐Sound quality	0.7894	0.8045	0.9575	0.6178	0.8504	0.7876	0.5969
A‐Frequency	0.6939	0.6826	0.9364	0.4500	0.7721	0.6932	0.4301
B‐Blood components	0.8250	0.8457	0.8017	0.8477	0.8198	0.8247	0.6552
B‐White blood cell	**0.8428**	**0.8678**	**0.8127**	**0.8726**	**0.8377**	**0.8427**	**0.6893**
B‐Enzyme	0.7067	0.6690	0.8201	0.5936	0.7361	0.7068	0.4265
B‐Electrolyte	0.5705	0.5614	0.7907	0.3480	0.6469	0.5694	0.1539
B‐Biochemistry	0.6749	0.6638	0.7244	0.6256	0.6904	0.6750	0.3539

*Note:* The bolded data in the table represent the feature group values with the best performance among the three modalities.

These features represent the respiratory stability, dynamic characteristics of the respiratory system, respiratory cycle patterns, respiratory duration and intensity, and immune system status of cancer patients, and have become the main information for predicting cancer. Through the aforementioned process of feature sorting, optimization, and evaluation, the extraction of key features that can represent various types of patient information was partially optimized, and in‐depth analysis was conducted. From the perspective of medical standardized blood modes, these highly contributing blood characteristics were highly consistent with the clinical criteria for judging lung cancer; however, problems such as insufficient reliability and insufficient access to information in judging lung cancer in a single mode were present. Therefore, from the two dimensions of macro respiratory vibration and respiratory sound combined with the blood dimension at the cellular and molecular levels, we jointly assist in diagnosing lung cancer, and have the multimodal advantage of comprehensively analyzing patients' lung cancer at the standardized level. Table 6 summarizes the representative features of the three modality optimizations.

**Table 6:** Representative features selected from each of the three modalities.

Nine frequency characteristic features in the fiber‐optic vibration modes:
Power spectrum maximum, Cepstrum peak factor, Fourier maximum, Cepstrum skewness, Cepstrum root mean square factor, Cepstrum signal‐to‐noise ratio, power spectrum signal‐to‐noise ratio, Fourier mean, Cepstrum impulse factor
Nine rhythmic features in the respiratory audio modality:
SPLmean, Emean, ZCR‐de‐range, STEsk, ZCR‐de‐min, ZCR‐de‐max, STEmin, STEku, SPL‐de‐range
Seven white blood cell‐related features in the blood index modality
Mononuclear cell ratio, basophil ratio, eosinophil count, white blood cell count, eosinophil ratio, basophil count, lymphocyte ratio

Abbreviations: de, first‐order difference; E, logarithmic energy value; ku, kurtosis; max, maximum; Min, minimum; sk, skewness; SPL, sound pressure level; STE, short‐term energy; ZCR, zero crossing rate.

#### 3.2.3 Feature Fusion

**Figure 11:**
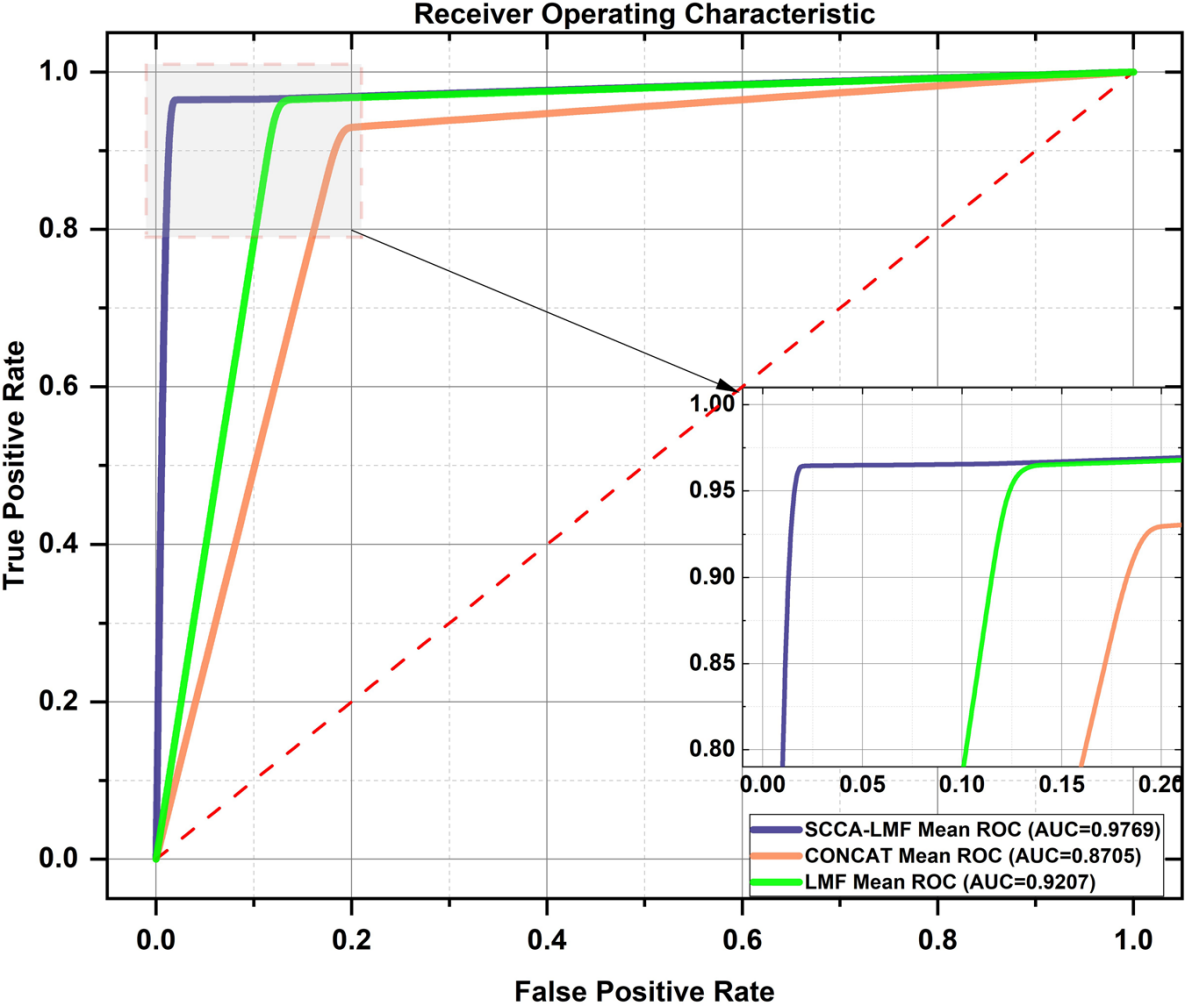
ROC curves for the three fusion methods.

The experimental results of optimizing the features of each of the three modalities showed that the comprehensive evaluation performance of the representative features of each modality was generally ideal, but shortcomings existed compared to the classification performance of all features of a single modality. In addition, considering the pathological limitations of each modality and the impact of external factors on data acquisition, such as fiber‐optic vibration and audio modalities, which are affected by environmental noise and unexpected body movements, hematological indicators may be affected by gender, weight, other infectious diseases, and medication use. Therefore, this study used the advantages of each of the three modalities' representative features to fuse and process the representative features of the three modalities' data. After fusing the three modalities' features, we compared the final results of the ensemble algorithm with the five classification methods selected during the algorithm process (Table 7). The ensemble learning algorithm significantly improved model performance compared to a single classification method. In the comparison of different feature fusion methods, this study used the SCCA‐LMF method, the LMF method, and the CONCAT method [24] from early fusion to fuse the preferred representative features of the three modalities and evaluate their performance. The experimental results are shown in Table 8, and the classification ROC curve is shown in Figure 11. SCCA‐LMF has a significant overall improvement in various performance indicators compared to traditional early fusion methods and LMF methods. This is because in the method that directly fuses respiratory audio signals with respiratory vibration signals using LMF or CONCAT, SCCA‐LMF first manually selects the representative features containing the most modality information using feature selection, achieving information concentration and filtering for the three modalities separately. Second, the SCCA method performs maximum mutual information processing on homologous respiratory‐related features, capturing the correlation between homologous and heterogeneous data, and further extracting the respiratory clinical features of lung cancer. Finally, the LMF method was used to tensor fuse the laboratory indicators and clinical features of heterologous lung cancer, enabling the fusion of feature information from both fields at the same latitude without losing either one.

**Table 7:** Comparison of lung cancer classification using five machine learning models.

Method	ACC	PERS	SENS	F1_S
XGboost	0.9645	0.9270	0.9924	0.9620
KNN	0.9027	0.9454	0.9285	0.9369
svm	0.9250	0.9545	0.8889	0.9142
LR	0.9588	0.9615	0.9565	0.9617
RF	0.9618	0.9615	0.9674	0.9644
**SCCA‐LMF**	**0.9770**	**0.9964**	**0.9575**	**0.9764**

**Table 8:** Evaluation metrics for the three fusion methods.

Design	ACC	PERS	SENS	SPEC	F1_S	AUC	MCC
LMF	0.9203	0.8852	**0.9642**	0.8771	0.9230	0.9207	0.8441
CONCAT	0.9027	0.9454	0.9285	0.8125	0.9369	0.8705	0.7254
SCCA‐LMF	**0.9770**	**0.9964**	0.9575	**0.9964**	**0.9764**	**0.9769**	**0.9550**

*Note:* The bold data in the table represent the best values of the three methods under seven performance indicators.

In the final results of the experiment, the specificity was 0.9964 and the sensitivity was 0.9575. However, a small number of samples were misdiagnosed in the experiment. In clinical practice, relevant research on the impact of lung diseases on respiratory mechanics in clinical medicine has shown that the impact of respiratory vibration on a few lung diseases, such as chronic obstructive pulmonary disease and tuberculosis, is similar to that of lung cancer. Therefore, our analysis of false positives can be summarized as follows: the presence of chronic obstructive pulmonary disease, tuberculosis, and other lung diseases in the data sample, together with lung cancer, affects the performance of the experiment in both fiber‐optic and audio macroscopic modes. Therefore, although the experimental model had high performance in diagnosing a single disease from a multimodal perspective, its compatibility with the combined effects of multiple diseases still needs improvement. Nevertheless, our misdiagnosis rate was controlled below 5%, ensuring overall good clinical diagnostic performance.

A detailed comparative analysis was conducted on several mainstream methods to further verify the advantages of the proposed lung disease prediction and evaluation method based on feature selection and multimodal fusion. The comparison results are summarized in Table 9.

**Table 9:** Comparison of the main methods used for lung cancer treatment and analysis.

Design	Reference	Sample	Sensitivity	Specificity	AUC
Imaging methods	Chen et al. [38]	69	0.85	0.85	0.93
	Xue et al. [39]	259	0.64	0.94	0.87
	Saied et al. [40]	1007	0.90	0.94	0.96
Biomarkers	Wu et al. [15]	226	0.96	0.95	0.99
	Fehlmann et al. [41]	3046	0.83	0.94	0.98
	Wang et al. [42]	429	0.91	0.83	0.95
Multimodal data fusion	Li et al. [43]	84	0.86	0.87	0.90
	Kumar et al. [44]	1156	—	0.94	—
	Our method	360	0.96	0.99	0.98

As shown in Table 9, the feature selection and multimodal fusion methods used in this study were compared with traditional imaging techniques and biomarker detection methods. In the same analysis of lung cancer samples, this article uses the premise of 360 samples combined with imbalanced data processing methods to process lung cancer samples. Finally, a high sensitivity of 0.96 and a high specificity of 0.99 were achieved, indicating a further improvement in the comprehensive indicators compared to single imaging methods or biomarker methods. This achievement not only demonstrates the effectiveness of the proposed method but also highlights its enormous potential in the preliminary prediction of lung cancer. Furthermore, in terms of multimodal data fusion, this study further improved the performance of the model by combining medical text data with respiratory sensing data. This fusion strategy not only enriched the dimensions of the data but also enhanced the model's understanding and ability to judge complex medical situations. The experimental results showed more significant advantages compared with other multimodal data fusion methods that rely solely on data sources, providing new research directions and ideas for the field of cancer prediction and evaluation.

## 4 Discussion

This study accurately predicts lung cancer and emphasizes its potential applications by integrating multimodal data: fiber optic respiratory vibration signals, respiratory audio signals, and routine hematological indicators, addressing key limitations of existing diagnostic methods. The excellent performance of the SCCA‐LMF method (97.70% accuracy, 95.75% sensitivity, and 99.64% specificity) is attributed to targeted high contribution feature selection and cross‐modal correlation capture, which outperforms traditional fusion methods such as single modal models and LMF. Unlike previous imaging or biomarker methods, it avoids radiation risks and high costs, making it suitable for resource‐limited areas.

It is worth noting that there are still certain limitations to the project results: only 360 sample queues from Gansu Province limit the universality of the region; A few other types of lung diseases produce respiratory vibrations similar to lung cancer, leading to false positives in the test; Although the interference of data collection environment and patient physical movement has been maximally limited in experimental design and algorithm, it still cannot completely eliminate the impact on the accuracy of experimental results. Future work will focus on expanding the regional sample, selectively filtering out the impact of other lung diseases on this experiment, and optimizing sensors to enhance the clinical practicality of the experimental model, while maintaining its core advantages in connecting engineering and healthcare in early large‐scale lung cancer screening.

## 5 Conclusions

In summary, this study used sorting classification selection fusion to optimize three‐modal data. The Smote algorithm was used to balance imbalanced sample data, and the SCCA‐LMF fusion method was used to achieve multimodal fusion of the three modalities, obtaining the optimal multimodal pathological fusion features for lung cancer. Afterward, Bayesian parameter tuning and accuracy ranking were used to optimize the hybrid ensemble learning algorithm. Finally, a mixed ensemble learning algorithm was used to assist in the prediction of the binary classification problem between lung cancer and nonlung cancer prediction. The predicted accuracy was 97.70%, sensitivity was 95.75%, and specificity was 99.64%. The cancer prediction research method based on feature optimization and multimodal fusion proposed in this study is beneficial for large‐scale screening and the preliminary prediction of cancer patients. During the data collection process, the friction between fiber‐optic cables and patient clothing, heart rate vibrations, and environmental noise are the main challenges faced by fiber‐optic and audio modal signal processing. We adopted DCT denoising technology to effectively filter out friction and heartbeat interference and reduce the impact of environmental noise using threshold detection and signal segmentation techniques. In addition, noninvasive blood collection, noncontact respiratory sound detection, and flexible fiber‐optic detection during the data acquisition process fully ensure the comfort of patients during the data collection process. The process is fast, simple, low‐cost, and can be integrated with current clinical testing methods, making it easy to use and promote. The use of machine learning to combine heterogeneous sensor data with traditional hematological indicators provides a new technique for predicting cancer risk populations on a large scale. In the future, further multimodal fusion of imaging data, multi‐omics data, age, smoking history, and the data presented here can be used to comprehensively evaluate lung cancer patients, while also having the ability to evaluate special individuals affected by complex diseases. However, since the patient samples used reflect patient data within Gansu Province centered on Lanzhou University Second Hospital, we will collect patient data from other regions to address the impact of regional factors on lung cancer patients.

## Author Contributions

**Jiawei Xu:** writing – original draft (equal). **Guodong Bao:** writing – original draft (equal). **Hansen Chen:** software (equal). **Yifan Zhao:** data curation (equal). **Mengqiang Yu:** data curation (equal). **Jiqiang Shang:** software (equal), validation (equal). **Yanxuan Luo:** investigation (equal), validation (equal). **Hongbo Ge:** investigation (equal), validation (equal). **Weiqi Hu:** investigation (equal), validation (equal). **Wenhua Zhang:** methodology (equal), resources (equal), supervision (equal), validation (equal). **Xiangyi Zan:** data curation (equal), formal analysis (equal), methodology (equal), project administration (equal), resources (equal), supervision (equal), validation (equal). **Zhixuan Yu:** resources (equal), supervision (equal). **Minjie Ma:** data curation (equal), formal analysis (equal), project administration (equal), resources (equal), supervision (equal), validation (equal). **Xiong Cao:** supervision (equal), validation (equal). **Menghao Guo:** investigation (equal), validation (equal). **Chenxi Shi:** investigation (equal), validation (equal). **Pengfei Cao:** data curation (equal), formal analysis (equal), funding acquisition (equal), investigation (equal), methodology (equal), project administration (equal), resources (equal), supervision (equal), writing – review and editing (equal). **Lin Cheng:** data curation (equal), project administration (equal), supervision (equal).

## Ethics Statement

The study was approved by the Ethics Committee of The Second Hospital of Lanzhou University, Lanzhou, China (No.2022A‐569).

## Consent

Informed written consent was obtained from all participants.

## Conflicts of Interest

The authors have nothing to report.

## Supporting information

Supplementary Documentation 2025.

## Data Availability

Data underlying the results presented in this paper are not publicly available at this time but may be obtained from the authors upon reasonable request. The underlying code for this study is not publicly available but may be made available to qualified researchers on reasonable request from the corresponding author. Data underlying the results presented in this paper are not publicly available at this time but may be obtained from the authors upon reasonable request. The underlying code for this study is not publicly available but may be made available to qualified researchers upon reasonable request from the corresponding author. The code and data obtained from the author can be used to reproduce all experimental results in the article.
