# Supplementary material for: Research on Cancer Prediction Based on Feature Optimization and Multimodal Fusion
Source: Health Care Sci. 2025 Dec 1;4(6):392–409. doi: 10.1002/hcs2.70040 (PMC12728679; doi:10.1002/hcs2.70040)
Supplement: Supplementary file 1 — Supplementary Documentation 2025. [file HCS2-4-392-s001.docx]

**FOSM Features-79 dimensions:**

| **Wavelet Feature Values (28)** | | | |
| --- | --- | --- | --- |
| D1 skewness | D1 kurtosis | D1 variance | D1 standard deviation |
| D2 skewness | D2 kurtosis | D2 variance | D2 standard deviation |
| D3 skewness | D3 kurtosis | D3 variance | D3 standard deviation |
| D4 skewness | D4 kurtosis | D4 variance | D4 standard deviation |
| D5 skewness | D5 kurtosis | D5 variance | D5 standard deviation |
| D6 skewness | D6 kurtosis | D6 variance | D6 standard deviation |
| D7 skewness | D7 kurtosis | D7 variance | D7 standard deviation |

| **Frequency Domain Feature Values (39)** | | |
| --- | --- | --- |
| cepstrum signal-to-noise ratio | Fourier signal-to-noise ratio | signal-to-noise ratio |
| cepstrum skewness | Fourier skewness | power spectrum median |
| cepstrum root mean square | Fourier root mean square | power spectrum signal-to-noise ratio |
| cepstrum root mean square factor | Fourier root mean square factor | power spectrum skewness |
| cepstrum peak factor | Fourier peak factor | power spectrum impulse factor |
| cepstrum kurtosis | Fourier kurtosis | power spectrum root mean square factor |
| cepstrum mean | Fourier mean | power spectrum peak factor |
| cepstrum mean absolute deviation | Fourier mean absolute deviation | power spectrum kurtosis |
| cepstrum variance | Fourier variance | power spectrum average |
| cepstrum maximum | Fourier maximum | power spectrum average power |
| cepstrum minimum | Fourier minimum | power spectrum average absolute deviation |
| cepstrum standard deviation | Fourier standard deviation | power spectrum maximum |
| cepstrum impulse factor |  | power spectrum minimum |
|  |  | power spectrum standard deviation |

| **Time Domain Feature Values (12)** | | |
| --- | --- | --- |
| mean value | mean absolute deviation | variance |
| maximum | minimum | standard deviation |
| root mean square | root mean square factor | peak factor |
| skewness | kurtosis | impulse factor |

**ASM Features-523 dimensions:**

| **Frequency Domain Feature Values-MFCC (273)** | | | | | | |
| --- | --- | --- | --- | --- | --- | --- |
| MFCC1min | MFCC1max | MFCC1range | MFCC1mean | MFCC1std | MFCC1sk | MFCC1ku |
| MFCC2min | MFCC2max | MFCC2range | MFCC2mean | MFCC2std | MFCC2sk | MFCC2ku |
| MFCC3min | MFCC3max | MFCC3range | MFCC3mean | MFCC3std | MFCC3sk | MFCC3ku |
| MFCC4min | MFCC4max | MFCC4range | MFCC4mean | MFCC4std | MFCC4sk | MFCC4ku |
| MFCC5min | MFCC5max | MFCC5range | MFCC5mean | MFCC5std | MFCC5sk | MFCC5ku |
| MFCC6min | MFCC6max | MFCC6range | MFCC6mean | MFCC6std | MFCC6sk | MFCC6ku |
| MFCC7min | MFCC7max | MFCC7range | MFCC7mean | MFCC7std | MFCC7sk | MFCC7ku |
| MFCC8min | MFCC8max | MFCC8range | MFCC8mean | MFCC8std | MFCC8sk | MFCC8ku |
| MFCC9min | MFCC9max | MFCC9range | MFCC9mean | MFCC9std | MFCC9sk | MFCC9ku |
| MFCC10min | MFCC10max | MFCC10range | MFCC10mean | MFCC10std | MFCC10sk | MFCC10ku |
| MFCC11min | MFCC11max | MFCC11range | MFCC11mean | MFCC11std | MFCC11sk | MFCC11ku |
| MFCC12min | MFCC12max | MFCC12range | MFCC12mean | MFCC12std | MFCC12sk | MFCC12ku |
| MFCC13min | MFCC13max | MFCC13range | MFCC13mean | MFCC13std | MFCC13sk | MFCC13ku |
| MFCC14min | MFCC14max | MFCC14range | MFCC14mean | MFCC14std | MFCC14sk | MFCC14ku |
| MFCC15min | MFCC15max | MFCC15range | MFCC15mean | MFCC15std | MFCC15sk | MFCC15ku |
| MFCC16min | MFCC16max | MFCC16range | MFCC16mean | MFCC16std | MFCC16sk | MFCC16ku |
| MFCC17min | MFCC17max | MFCC17range | MFCC17mean | MFCC17std | MFCC17sk | MFCC17ku |
| MFCC18min | MFCC18max | MFCC18range | MFCC18mean | MFCC18std | MFCC18sk | MFCC18ku |
| MFCC19min | MFCC19max | MFCC19range | MFCC19mean | MFCC19std | MFCC19sk | MFCC19ku |
| MFCC20min | MFCC20max | MFCC20range | MFCC20mean | MFCC20std | MFCC20sk | MFCC20ku |
| MFCC21min | MFCC21max | MFCC21range | MFCC21mean | MFCC21std | MFCC21sk | MFCC21ku |
| MFCC22min | MFCC22max | MFCC22range | MFCC22mean | MFCC22std | MFCC22sk | MFCC22ku |
| MFCC23min | MFCC23max | MFCC23range | MFCC23mean | MFCC23std | MFCC23sk | MFCC23ku |
| MFCC24min | MFCC24max | MFCC24range | MFCC24mean | MFCC24std | MFCC24sk | MFCC24ku |
| MFCC25min | MFCC25max | MFCC25range | MFCC25mean | MFCC25std | MFCC25sk | MFCC25ku |
| MFCC26min | MFCC26max | MFCC26range | MFCC26mean | MFCC26std | MFCC26sk | MFCC26ku |
| MFCC27min | MFCC27max | MFCC27range | MFCC27mean | MFCC27std | MFCC27sk | MFCC27ku |
| MFCC28min | MFCC28max | MFCC28range | MFCC28mean | MFCC28std | MFCC28sk | MFCC28ku |
| MFCC29min | MFCC29max | MFCC29range | MFCC29mean | MFCC29std | MFCC29sk | MFCC29ku |
| MFCC30min | MFCC30max | MFCC30range | MFCC30mean | MFCC30std | MFCC30sk | MFCC30ku |
| MFCC31min | MFCC31max | MFCC31range | MFCC31mean | MFCC31std | MFCC31sk | MFCC31ku |
| MFCC32min | MFCC32max | MFCC32range | MFCC32mean | MFCC32std | MFCC32sk | MFCC32ku |
| MFCC33min | MFCC33max | MFCC33range | MFCC33mean | MFCC33std | MFCC33sk | MFCC33ku |
| MFCC34min | MFCC34max | MFCC34range | MFCC34mean | MFCC34std | MFCC34sk | MFCC34ku |
| MFCC35min | MFCC35max | MFCC35range | MFCC35mean | MFCC35std | MFCC35sk | MFCC35ku |
| MFCC36min | MFCC36max | MFCC36range | MFCC36mean | MFCC36std | MFCC36sk | MFCC36ku |
| MFCC37min | MFCC37max | MFCC37range | MFCC37mean | MFCC37std | MFCC37sk | MFCC37ku |
| MFCC38min | MFCC38max | MFCC38range | MFCC38mean | MFCC38std | MFCC38sk | MFCC38ku |
| MFCC39min | MFCC39max | MFCC39range | MFCC39mean | MFCC39std | MFCC39sk | MFCC39ku |
| *min : minimum ; max : maximum ; std : standard deviation ; sk : skewness ; ku : kurtosis* | | | | | | |

| **Timbre Features Values (130)** | | | | | | |
| --- | --- | --- | --- | --- | --- | --- |
| F1min | F1max | F1range | F1mean | F1std | F1sk | F1ku |
| F2min | F2max | F2range | F2mean | F2std | F2sk | F2ku |
| F3min | F3max | F3range | F3mean | F3std | F3sk | F3ku |
| F1-de-min | F1-de-max | F1-de-range | F1-de-mean | F1-de-std | F1-de-sk | F1-de-ku |
| F2-de-min | F2-de-max | F2-de-range | F2-de-mean | F2-de-std | F2-de-sk | F2-de-ku |
| F3-de-min | F3-de-max | F3-de-range | F3-de-mean | F3-de-std | F3-de-sk | F3-de-ku |
| F1-de2-min | F1-de2-max | F1-de2-range | F1-de2-mean | F1-de2-std | F1-de2-sk | F1-de2-ku |
| F2-de2-min | F2-de2-max | F2-de2-range | F2-de2-mean | F2-de2-std | F2-de2-sk | F2-de2-ku |
| F3-de2-min | F3-de2-max | F3-de2-range | F3-de2-mean | F3-de2-std | F3-de2-sk | F3-de2-ku |
| B1min | B1max | B1range | B1mean | B1std | B1sk | B1ku |
| B2min | B2max | B2range | B2mean | B2std | B2sk | B2ku |
| B3min | B3max | B3range | B3mean | B3std | B3sk | B3ku |
| B1-de-min | B1-de-max | B1-de-range | B1-de-mean | B1-de-std | B1-de-sk | B1-de-ku |
| B2-de-min | B2-de-max | B2-de-range | B2-de-mean | B2-de-std | B2-de-sk | B2-de-ku |
| B3-de-min | B3-de-max | B3-de-range | B3-de-mean | B3-de-std | B3-de-sk | B3-de-ku |
| B1-de2-min | B1-de2-max | B1-de2-range | B1-de2-mean | B1-de2-std | B1-de2-sk | B1-de2-ku |
| B2-de2-min | B2-de2-max | B2-de2-range | B2-de2-mean | B2-de2-std | B2-de2-sk | B2-de2-ku |
| B3-de2-min | B3-de2-max | B3-de2-range | B3-de2-mean | B3-de2-std | B3-de2-sk | B3-de2--ku |
| absolute frequency perturbation | | | relative frequency perturbation | | | |
| absolute amplitude perturbation | | | relative amplitude perturbation | | | |
| *min : minimum ; max : maximum ; std : standard deviation ; sk : skewness ; ku : kurtosis*  *de : First order difference ; de2 : Second order difference*  *F1/F2/F3 : Center frequency of resonance peak ; B1/B2/B3 : The bandwidth at the resonance peak's center frequency.* | | | | | | |

| **Melodic Features Values (120)** | | | | | | |
| --- | --- | --- | --- | --- | --- | --- |
| VD-min | VD-max | VD-range | VD-mean | VD-std |  |  |
| VS-min | VS-max | VS-range | VS-mean | VS-std |  |  |
| ESS-min | ESS-max | ESS-range | ESS-mean | ESS-std |  |  |
| F0min | F0max | F0range | F0mean | F0std | F0sk | F0ku |
| F0-de-min | F0-de-max | F0-de-range | F0-de-mean | F0-de-std | F0-de-sk | F0-de-ku |
| F0-de2-min | F0-de2-max | F0-de2-range | F0-de2-mean | F0-de2-std | F0-de2-sk | F0-de2-ku |
| Emin | Emax | Erange | Emean | Estd | Esk | Eku |
| E-de-min | E-de-max | E-de-range | E-de-mean | E-de-std | E-de-sk | E-de-ku |
| E-de2-min | E-de2-max | E-de2-range | E-de2-mean | E-de2-std | E-de2-sk | E-de2-ku |
| STEmin | STEmax | STErange | STEmean | STEstd | STEsk | STEku |
| STE-de-min | STE-de-max | STE-de-range | STE-de-mean | STE-de-std | STE-de-sk | STE-de-ku |
| STE-de2-min | STE-de2-max | STE-de2-range | STE-de2-mean | STE-de2-std | STE-de2-sk | STE-de2-ku |
| ZCRmin | ZCRmax | ZCRrange | ZCRmean | ZCRstd | ZCRsk | ZCRku |
| ZCR-de-min | ZCR-de-max | ZCR-de-range | ZCR-de-mean | ZCR-de-std | ZCR-de-sk | ZCR-de-ku |
| ZCR-de2-min | ZCR-de2-max | ZCR-de2-range | ZCR-de2-mean | ZCR-de2-std | ZCR-de2-sk | ZCR-de2-ku |
| SPLmin | SPLmax | SPLrange | SPLmean | SPLstd | SPLsk | SPLku |
| SPL-de-min | SPL-de-max | SPL-de-range | SPL-de-mean | SPL-de-std | SPL-de-sk | SPL-de-ku |
| SPL-de2-min | SPL-de2-max | SPL-de2-range | SPL-de2-mean | SPL-de2-std | SPL-de2-sk | SPL-de2-ku |
| *VD : Voiced voice ; VS : Voiceless ; ESS : Effective speech segments*  *F0 : Fundamental frequency ; E : Logarithmic energy value ; STE : Short term energy ; ZCR : Zero crossing rate ; SPL : Sound pressure level*  *de : First order difference ; de2 : Second order difference* | | | | | | |

**ROC curves of three auxiliary diagnostic modalities for lung cancer:**


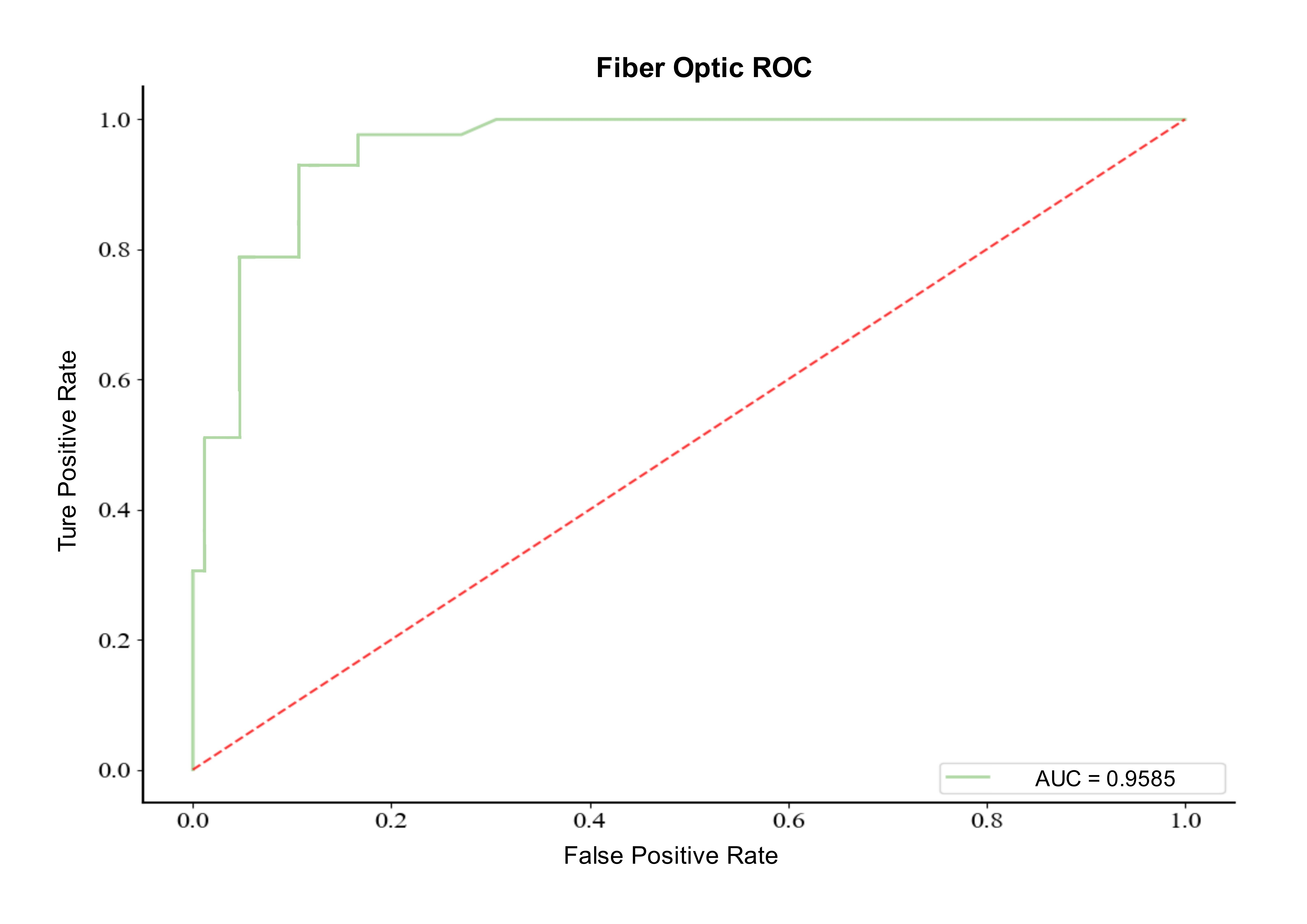


**Fiber optic modal ROC curve**


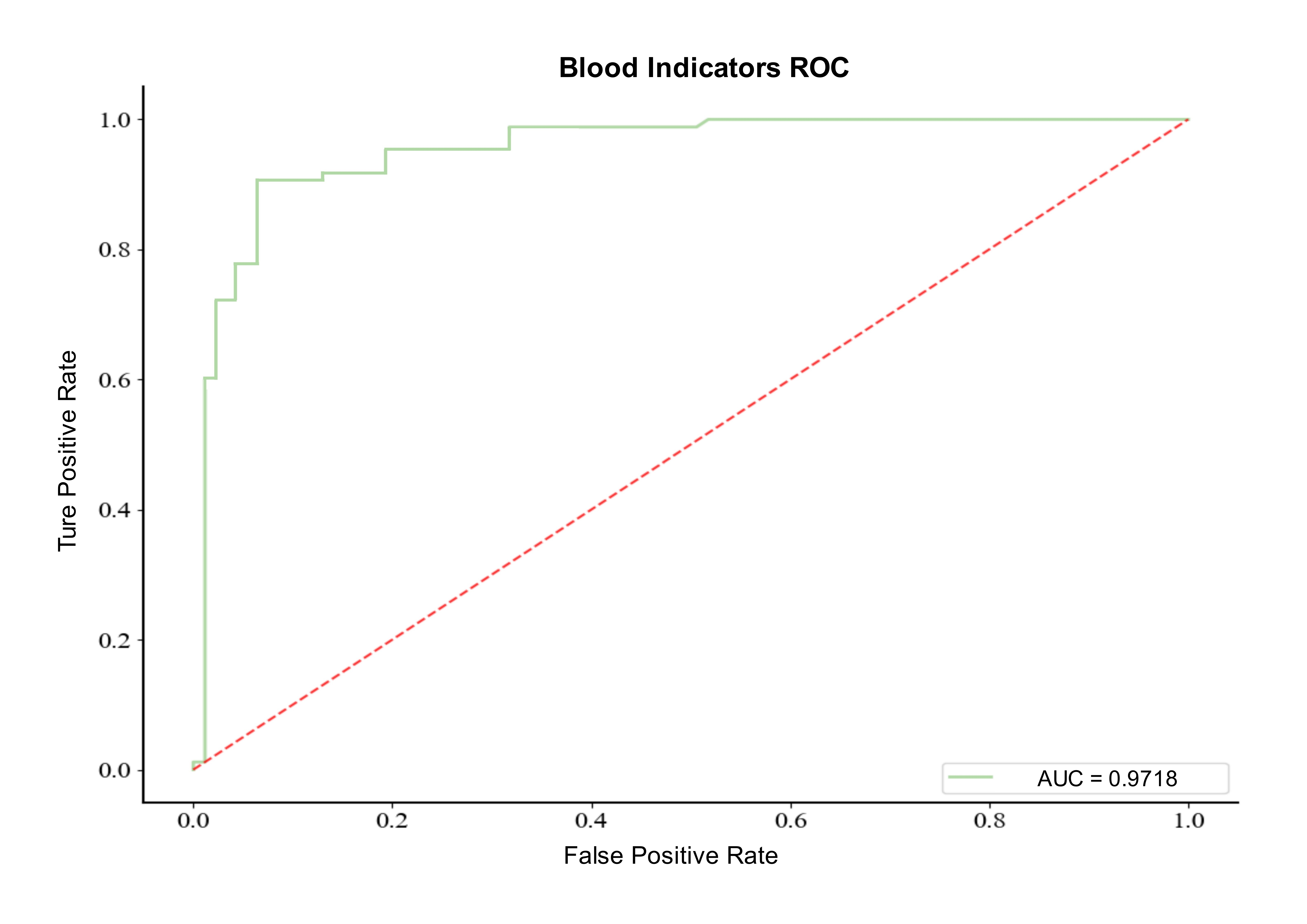


**Blood modal ROC curve**


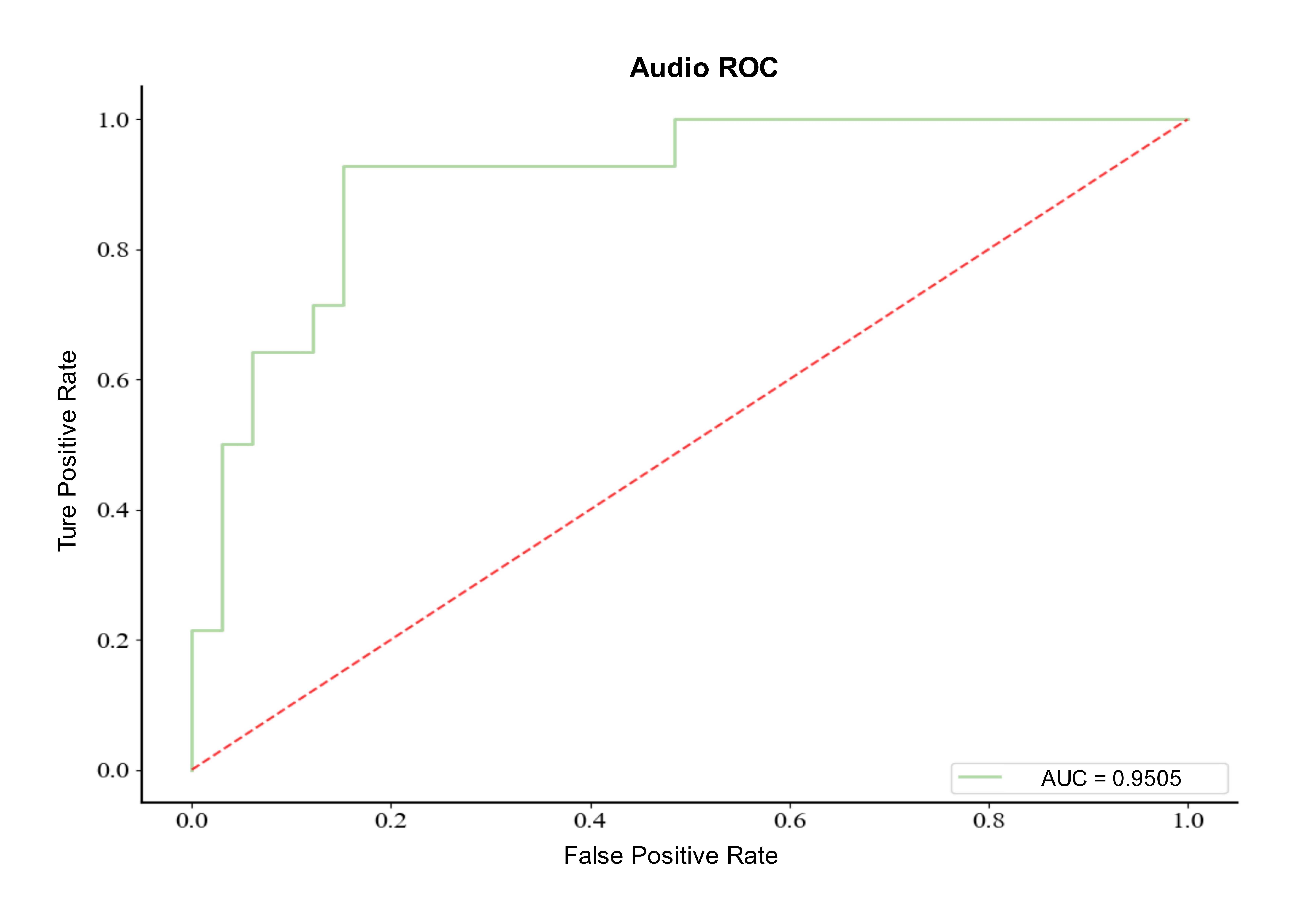


**Audio modal ROC curve**

**ROC curve of SCCA-LMF algorithm:**

**
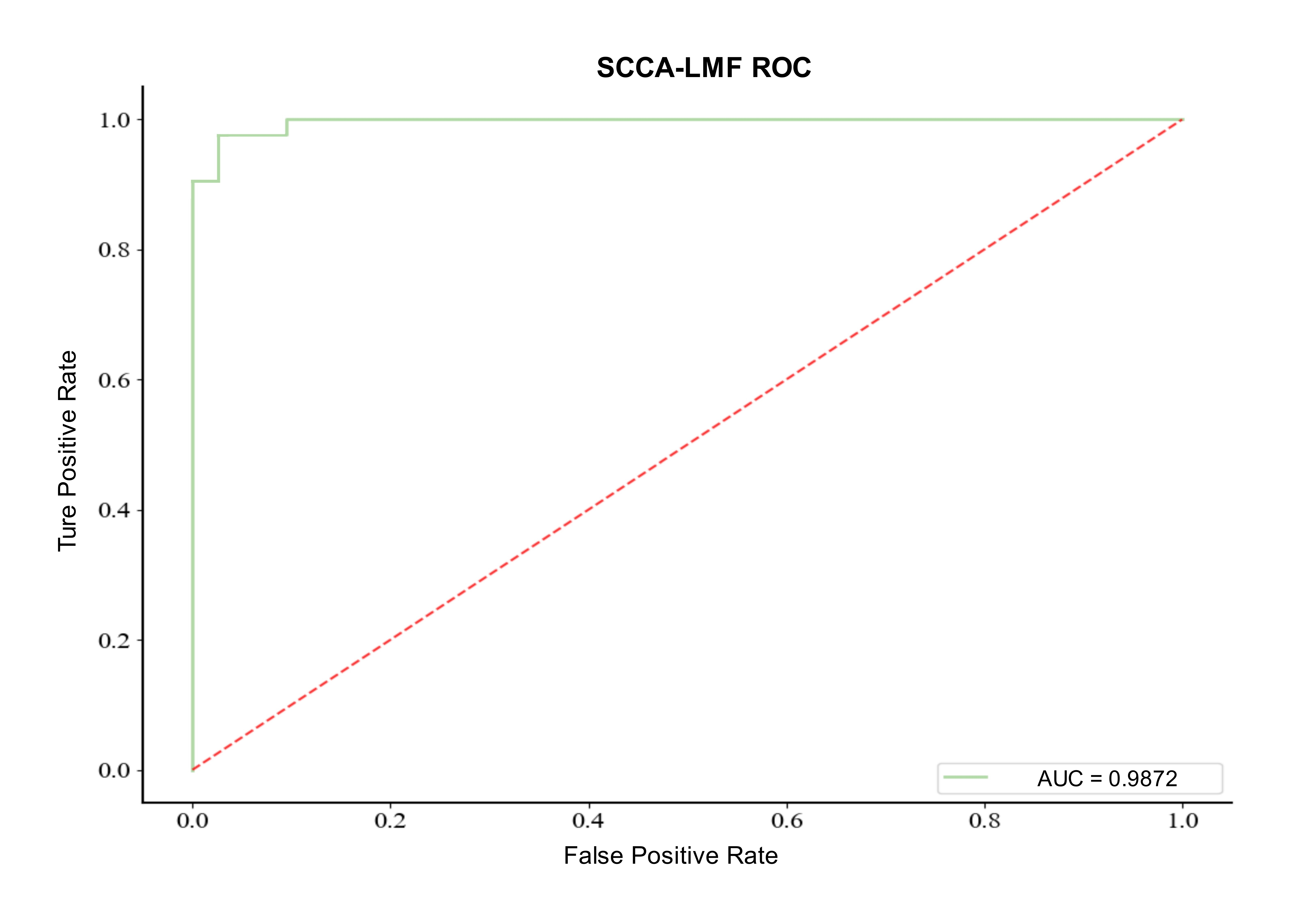
**

**SCCA-LMF ROC curve**
